# A proteinaceous organic matrix regulates carbonate mineral production in the marine teleost intestine

**DOI:** 10.1038/srep34494

**Published:** 2016-10-03

**Authors:** Kevin L. Schauer, Christophe M. R. LeMoine, Adrian Pelin, Nicolas Corradi, Wesley C. Warren, Martin Grosell

**Affiliations:** 1 Marine Biology and Ecology, Rosenstiel School of Marine and Atmospheric Science, University of Miami, Miami, FL, 33149, USA; 2 Department of Biology, Brandon University, Brandon, MB, R7A 6A9, Canada; 3 Canadian Institute for Advanced Research, Department of Biology, University of Ottawa, Ottawa, ON, K1N 6N5, Canada; 4 McDonnell Genome Institute, Washington University School of Medicine, St Louis, Missouri 63108, USA

## Abstract

Marine teleost fish produce CaCO_3_ in their intestine as part of their osmoregulatory strategy. This precipitation is critical for rehydration and survival of the largest vertebrate group on earth, yet the molecular mechanisms that regulate this reaction are unknown. Here, we isolate and characterize an organic matrix associated with the intestinal precipitates produced by Gulf toadfish (*Opsanus beta*). Toadfish precipitates were purified using two different methods, and the associated organic matrix was extracted. Greater than 150 proteins were identified in the isolated matrix by mass spectrometry and subsequent database searching using an *O. beta* transcriptomic sequence library produced here. Many of the identified proteins were enriched in the matrix compared to the intestinal fluid, and three showed no substantial homology to any previously characterized protein in the NCBI database. To test the functionality of the isolated matrix, a micro-modified *in vitro* calcification assay was designed, which revealed that low concentrations of isolated matrix substantially promoted CaCO_3_ production, where high concentrations showed an inhibitory effect. High concentrations of matrix also decreased the incorporation of magnesium into the forming mineral, potentially providing an explanation for the variability in magnesium content observed in precipitates produced by different fish species.

Marine teleosts live in a hyperosmotic environment and therefore must constantly compensate for passive water loss and ion gain. To avoid dehydration, these fish must extract water from the only source available: seawater. The high osmolality of seawater (>1000 mOsm/kg) compared to that of fish extracellular fluids (300–350 mOsm/kg) makes the uptake of water challenging. Most of the water absorption that occurs across in the gastrointestinal tract does so across the intestinal epithelium, despite the lack of any substantial osmotic gradient^1^. Water is instead moved by solute-coupled water transport, where the net absorption of monovalent ions (namely Na^+^ and Cl^−^) across the epithelium drives concurrent water uptake^2^. In all, 60–85% of the imbibed water is absorbed by the intestine in this fashion^3^. Conversely, the intestinal epithelium is largely impermeable to divalent ions such as magnesium, sulfate and calcium, which are also relatively abundant in seawater (approximately 50 mM, 30 mM and 10 mM, respectively)^4^. The high rates of water absorption along the gastrointestinal tract lead to the several-fold concentration of these ions, with sulfate and magnesium concentrations in the rectal fluid exceeding 100 mM^5^. It is the concentration of these ions that ultimately limits the ability of water uptake (and hence osmoregulatory ability) in these fish during hypersalinity exposure^4^,^5^. Interestingly, the luminal calcium concentrations do not show this trend and are usually less than 5 mM in the rectal fluid as opposed to the 30–50 mM that would be expected due to the rate of water absorption^4^,^5^. This discrepancy is due to the precipitation of CaCO_3_ that occurs in the intestinal lumen. Precipitation occurs due to alkalization (pH 8.3 to 9.2) of the intestinal fluid by bicarbonate transport (primarily in the anterior intestine) into the lumen in exchange for chloride uptake^1^,^4^,^6^,^7^. The precipitation of CaCO_3_ lowers the luminal osmolality by as much as 100 mOsm/kg by lowering the concentrations of Ca^2+^ and HCO_3_^−^ in solution, which aids in water absorption^4^. The CaCO_3_ produced is eventually excreted to the environment as a waste product. Without the reduced osmolality in the intestinal lumen resulting from this precipitation reaction, teleosts would be unable to inhabit marine environments, as they would be unable to sufficiently rehydrate^8^.

Due to the large biomass, as well as high individual rates of carbonate production and excretion, a conservatively estimated 40 to 110 × 10^6^ metric tons of CaCO_3_ is produced by marine teleosts and excreted into the environment each year, which accounts for approximately 3 to 15% of the total oceanic CaCO_3_ production^6^. In most instances, these carbonates contain high amounts of Mg, which make them more soluble than other marine derived carbonate minerals such as aragonite^9^,^10^. It has been suggested that the dissolution of these highly soluble carbonates may be responsible for the observed increase in total alkalinity above the aragonite saturation horizon that is observed in oceanic surface waters^9^. High-Mg calcites, such as those produced by fish, are also anticipated to play an important role in the early response to ocean acidification caused by anthropogenic CO_2_ release^11^.

Despite the importance of piscine carbonate production to marine fish survival and the oceanic carbon cycle, little is known about how the precipitation reaction is controlled *in vivo*. The ion transport mechanisms that produce an environment favorable for the precipitation of CaCO_3_ in the intestine have been well-studied^1^,^4^,^12^, but the molecular mechanisms regulating the precipitation reaction itself have not yet been investigated. In all other well-studied eukaryote biomineralization processes, the inorganic mineral is deposited in association with an organic, typically primarily proteinaceous matrix^13^,^14^,^15^. This organic matrix is often involved in controlling the rate of precipitation, as well as the structure and composition of the resulting mineral^16^,^17^. Although the composition of the matrix varies significantly among species^15^,^18^ and even the different minerals formed within the same species^19^, several general characteristics are shared between the proteins found in many different biomineralization systems. Perhaps most common, especially in instances where calcium-containing minerals are formed, is the presence of highly acidic proteins in the mineral matrix^13^,^20^,^21^. The acidic nature of these proteins stems from both a high aspartic and glutamic acid composition, as well as the presence of negatively charged post-translational modifications such as phosphorylation or glycosylation^13^. The anionic characteristics of these macromolecules allows for their interaction with the cationic constituents of the forming mineral (such as Ca^2+^), which have been demonstrated to both enhance, and inhibit mineral formation depending on the situation^22^. Proteins involved in biomineralization are also often highly disordered, with large portions of the protein lacking significant secondary or tertiary structure^18^. These intrinsically disordered proteins are believed to play a major role in the inhibition of unwanted excess mineralization, as their flexibility and lack of defined structure provides a larger binding surface for which the protein can bind to already formed mineral step edges, inhibiting further growth^18^,^23^.

Early reports suggested the presence of an organic matrix in the intestinal-derived piscine carbonates^24^, but to date no research has been conducted to confirm or characterize such a matrix. In the present study, we purify intestinal precipitates produced by the Gulf toadfish (*Opsanus beta*) and identify >150 proteins that are associated with these precipitates. We then demonstrate that these isolated proteins modulate the rate of CaCO_3_ production *in vitro*, as well as the Mg incorporation into the resulting mineral, in a highly dose-dependent, but not straightforward manner.

## Results

### Purification of intestinal precipitates and extraction of organic matrix

In order to determine if the intestinal precipitates formed in marine fish contain an organic matrix, the CaCO_3_ precipitates were purified by two different methods in order to remove intestinal contaminants that could be associated with the mineral. First, a gentle purification procedure was designed to keep the majority of the internal infrastructure of the precipitates intact, while removing the thick mucus layer that covers the precipitates *in vivo*. Second, a stringent purification procedure was intended to remove all organic material that was not completely imbedded in the CaCO_3_ mineral. Precipitates purified using the gentle procedure retained the majority of their ultrastucture, whereas those purified by the stringent method were reduced to a predominantly fine powder. After purification, precipitates prepared using each of the procedures, as well as unpurified precipitates, were imaged by scanning electron microscopy (SEM) to observe if the mucus layer and other large contaminants had been removed from the mineral. SEM analysis revealed a thick mucilaginous layer covering the majority of the unpurified precipitates, while no mucus was observed on the CaCO_3_ that was purified by either method (Supplementary Fig. S1).

**Figure 1:**
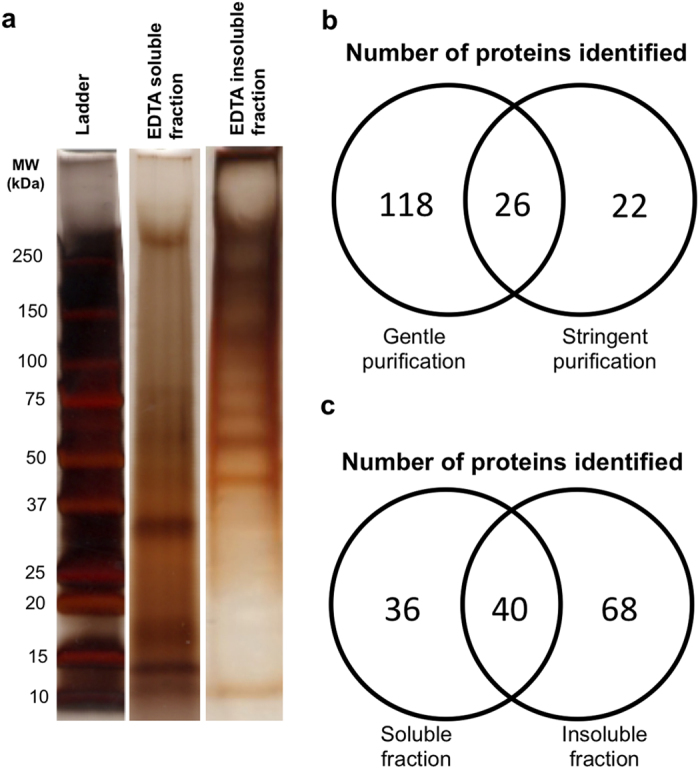
Identification of a proteinaceous matrix isolated from intestinally derived CaCO_3_ precipitates. (**a**) Cropped lanes from silver stained SDS-PAGE gels showing proteins isolated from CaCO_3_ precipitates extracted directly from the intestine of Gulf toadfish (*Opsanus beta*) and purified by the gentle purification method. Proteins were divided into two fractions based on their solubility in EDTA. Uncropped gel images can be found in Supplementary Fig. S9. (**b**) Comparison of the number of proteins identified by mass spectrometry between the gentle and stringent purification procedures. (**c**) The number of proteins identified using mass spectrometry in the EDTA soluble and EDTA insoluble fractions extracted from precipitates purified using the gentle purification procedure.

The inorganic mineral was then removed from the purified precipitates by incubation in a solution of 0.5 M ethylenediaminetetraacetic acid (EDTA). The resulting solution contained any organic matrix that was originally associated with the mineral. In precipitates purified using the gentle purification procedure, insoluble material (likely consisting of non-water soluble proteins) was clearly visible in the EDTA solution even after the CaCO_3_ mineral was completely dissolved. This resulting EDTA solution was therefore centrifuged, and the insoluble pellet was resolubilized in an 8 M urea solution and termed the EDTA insoluble fraction, where the supernatant was classified as the EDTA soluble fraction. In precipitates purified by the stringent purification procedure, no pellet was observed after centrifugation of the EDTA solution, so no insoluble fraction was collected. Both fractions collected from gently purified samples were analyzed by SDS-PAGE and silver stained to determine if proteins were present (Fig. 1a). Numerous bands were clearly visible at a wide variety of molecular weights in both fractions, suggesting the presence of an at least partially proteinaceous organic matrix associated with the intestinally derived CaCO_3_ precipitates.

### Gulf toadfish transcriptome

In order to allow for subsequent protein identification by mass spectrometry (MS), next-generation sequencing was used to obtain transcriptomic sequence information from the Gulf toadfish (described in detail in the Supplementary Methods). RNA extracted from numerous tissues (Supplementary Table S1) that originated from a single *Opsanus beta* individual were subjected to Illumina RNA deep sequencing, which resulted in an average of 93 M (±2 M) reads per sample. These were assembled using Oases^25^ into an average of 19,389 (±5,504) mRNA loci with an average of 1.34 isoforms per locus. Within each library, there was on average 3,212 (±847) unique transcripts, of which 40.1% (±5.3) had homologies with sequences in the NCBI databases and 15% (±15.2) could be assigned GO terms. Overall, the GO term enrichment patterns support the uniqueness of each tissue type in terms of the transcript abundance with respect to their function. Of particular interest to the present study, gastro-intestinal tissues presented high abundance of genes associated with proteolysis, transmembrane processes, transport, and carbohydrate metabolism (Supplementary Fig. S2). Similarly, metabolically active tissues such as muscles had an overrepresentation of genes involved in redox processes, metabolic processes, as well as protein and ATP binding (Supplementary Fig. S2). All assemblies and raw reads were made available on NCBI under BioProject PRJNA313355.

### Mass spectrometry analysis of isolated precipitate matrix and intestinal fluid

Identities of the proteins associated with the intestinal precipitates were determined by MS analysis of both the gentle and stringent purified precipitate matrix. As anticipated, more unique proteins were identified in samples purified by the gentle purification (144) than in those processed by the stringent purification method (48; Fig. 1b). Of those proteins identified in the gently purified matrix samples, 68 were observed only in the insoluble fractions, where 36 were unique to the soluble fraction. Complete listings of all proteins identified in the MS analysis are available in Supplementary Tables S2 and S3. Interestingly 14 of the proteins identified in gently purified matrix contain known calcium binding domains (as determined using the NCBI conserved domain search^26^), where only two were observed in the stringently purified samples (Supplementary Tables S2 and S3).

**Figure 2:**
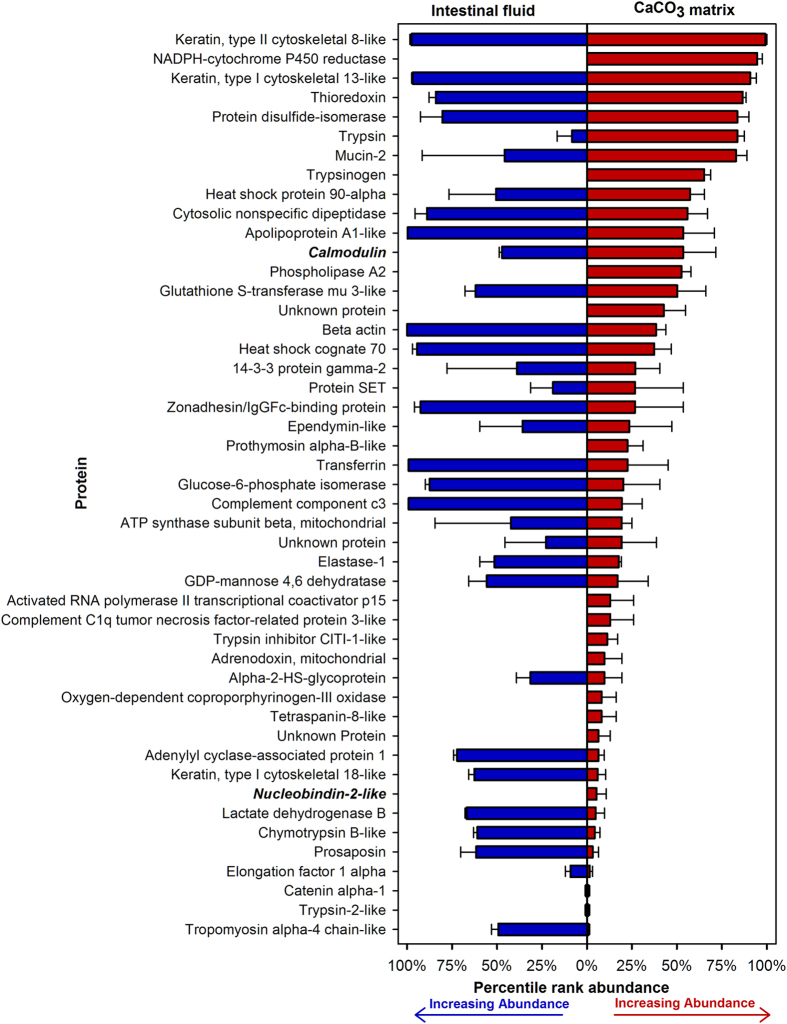
Relative abundance of proteins identified in the matrix (red) of precipitates purified using the stringent purification procedure as compared to raw intestinal fluid (blue). The number of spectral counts (SpC) assigned to each protein during mass spectrometry analysis was used as a proxy of relative protein abundance. The percentile rank in each protein was determined for each sample and plotted to highlight differences in composition in the intestinal fluid as compared to the purified CaCO_3_ matrix. Values are mean percentile rank ± SEM (n = 2 for intestinal fluid samples; n = 3 for CaCO_3_ matrix samples). Proteins in bold and italics contain known calcium binding domains as determined by the NCBI conserved domain database.

To compare the proteins observed in the isolated matrix to those found in the intestinal fluid, whole intestinal fluid protein extracts were also analyzed. A total of 509 unique proteins were identified in the intestinal fluid (Supplementary Table S3). In order to determine if the proteins identified in the precipitate matrix were simply intestinal contaminants, or if certain proteins were enriched in the matrix, the relative percentile rank abundance of each protein was calculated in the intestinal fluid and stringently purified matrix samples. The number of spectral counts (SpC) assigned to each protein was used to estimate protein abundance, with higher numbers of SpC suggesting higher abundance^27^. When these percentile ranks are compared (Fig. 2), several proteins are clearly enriched in the matrix compared to the intestinal fluid. Additionally, 13 proteins were present in precipitate matrix but were not detected in the intestinal fluids. Further, the majority of the proteins identified in the matrix are not known calcium-binding proteins, suggesting that this enrichment is not due to nonspecific association of calcium-binding proteins to the high concentration of calcium in the precipitates.

### Effect of isolated matrix on the rate of CaCO_3_ formation *in vitro*

**Figure 3:**
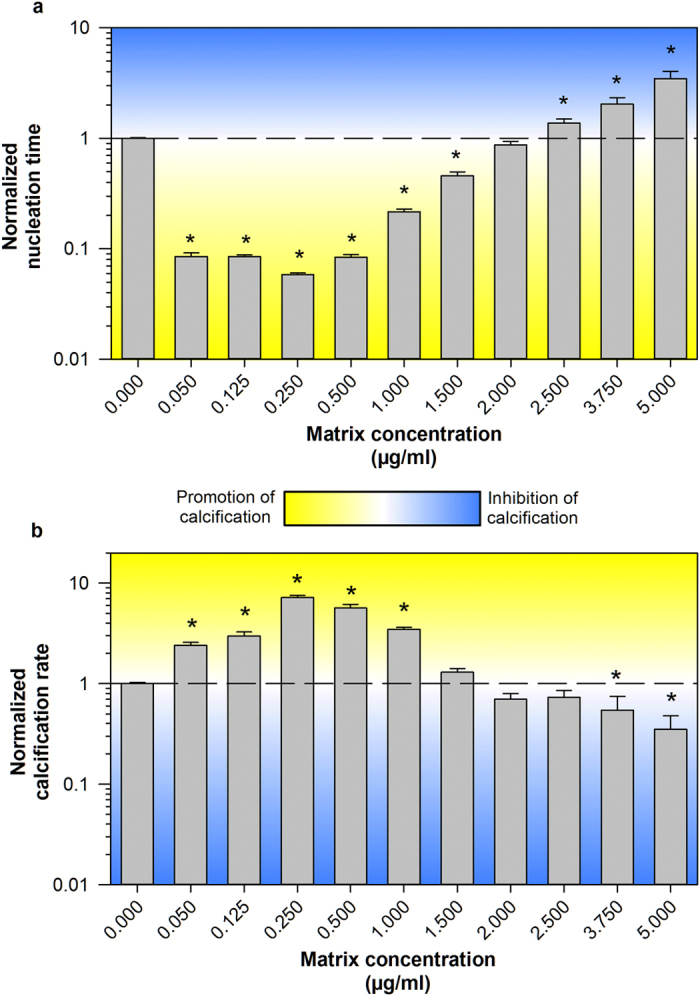
The effect of varying concentrations of isolated CaCO_3_ matrix on the rate of CaCO_3_ production *in vitro*. No protein control normalized nucleation time (**a**) and calcification rate (**b**). Matrix extracted from CaCO_3_ precipitates purified using the stringent purification procedure was analyzed using the micro-modified pH-drift calcification assay. Protein concentrations are reported as the final concentration of protein in the microplate well. Areas shaded in blue are indicative of inhibition of CaCO_3_ production, where those shaded yellow represent increased CaCO_3_ production. Values are normalized to the no protein controls and are represented as mean ± SEM (n = 6 except for the 5.0 μg/ml sample where only 4 of the 6 replicates showed any precipitation of CaCO_3_ during the assay). Asterisks indicate values that are significantly (p < 0.05) different from the no protein control as determined by two-sided ANOVA of log-transformed data, followed by multiple comparisons via the Holm-Sidak method.

A micro-modified *in vitro* calcification assay (described in detail in the Methods section) was designed in order to monitor the production of CaCO_3_ in the presence of varying concentrations of matrix over time. This method allowed for two metrics of CaCO_3_ production (nucleation time and calcification rate; Supplementary Fig. S3) to be calculated for each sample tested, allowing for inter-sample comparison. Stringently purified precipitate matrix was subjected to the calcification assay at varying concentration to determine its effect on calcification. A strong, dose-dependent response was observed for both nucleation time and calcification rate (Fig. 3). At low matrix protein concentrations, a significant increase in CaCO_3_ production (decrease in nucleation time, increase in calcification rate) was observed compared to the no protein controls. Interestingly, the trend diminished as protein concentration increased, and eventually reversed (nucleation time was significantly increased and calcification rate was decreased compared to controls) at higher concentrations. Additionally, at 5.0 μg/ml, only four of the six replicates showed a substantial drop in pH, suggesting that CaCO_3_ production was completely inhibited in the other two samples. The calcification assay was also conducted using varying concentrations of bovine serum albumin (BSA) to ensure the observed effects were not due to some general protein effect. No change in CaCO_3_ production was observed in the presence of low concentrations of BSA, but a small inhibitory effect (increase in nucleation time, decrease in calcification rate) was observed with the addition of 1.0 and 2.5 μg/ml BSA (Supplementary Fig. S4). The change in both parameters was less than 75% however, and therefore, substantially less that what was observed with the matrix.

In order to confirm the results from the newly developed calcification assay, several concentrations of matrix were retested using an alternative, pH-stat method^28^ (described in detail in the Supplementary Methods). This method maintains a constant pH in the calcifying solution by the addition of NaOH, and the rate of NaOH addition can be once again used to calculate nucleation times and calcification rates (Supplementary Fig. S5). Similar to the results observed in the micro-modified calcification assay, a decrease in nucleation time was observed when low concentrations of matrix were present (suggesting increased CaCO_3_ production), where an increase in nucleation time (and decrease in CaCO_3_ production) was observed at higher concentrations as compared to the no protein controls (Supplementary Fig. S6). Thus, the pH-stat assay verified the calcification assay measurements. However, it should be noted that the concentrations at which these effects were observed were lower than with the calcification assay. Interestingly, no significant changes in calcification rate were found across any of the samples (Supplementary Fig. S6).

### Effect of precipitate matrix on the incorporation of magnesium into the formed mineral

**Figure 4:**
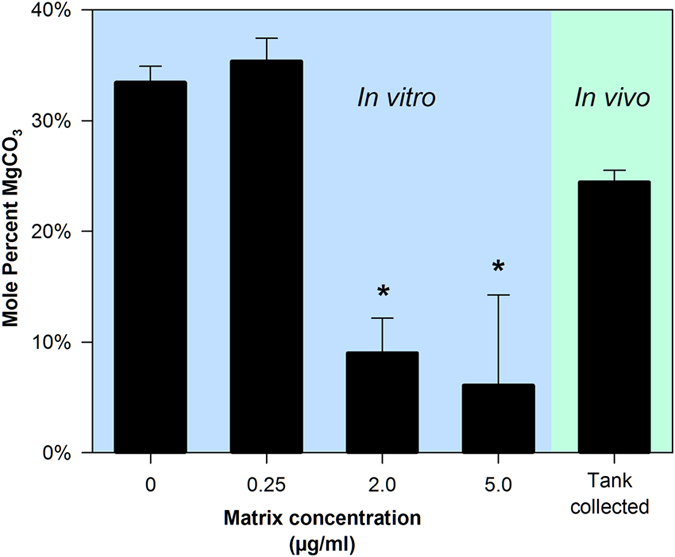
Effect of isolated intestinal precipitate matrix on the Mg:Ca ratio of CaCO_3_ produced *in vitro*. Matrix extracted from intestinal precipitates purified using the stringent purification procedure was added at varying concentrations to a saline that mimics the ion composition found in the teleost intestinal lumen. Formed precipitates were analyzed by energy dispersive spectroscopy (EDS) to determine the magnesium to calcium ratio. Samples in the blue shaded area were produced *in vitro* using varying concentrations of matrix, where the samples in the green area were produced by a fish and collected from the tank after excretion. The *in vivo* produced samples were purified using NaOCl to remove organic matter. Values represent mean percent MgCO_3_ ± SEM (n = 3). Asterisks indicate values that differ significantly (p < 0.05) from the no protein control as determined by two-sided ANOVA and multiple comparison using the Holm-Sidak method.

Large variations in the Mg content of fish-derived intestinal precipitates have been observed across species^10^,^29^,^30^. In order to determine if this observed variation could be due to differences in the organic matrix produced by different species, we investigated the role of isolated matrix on the Mg content of *in vitro* produced CaCO_3_. Scanning electron microscopy (SEM) and energy dispersive spectroscopy (EDS) was used to observe the morphology and determine the Mg:Ca ratio, respectively, of *in vitro* produced CaCO_3_ formed in the presence of varying concentrations of isolated precipitate matrix. The presence of isolated matrix did not appear to have a substantial effect on the crystal morphology of the formed CaCO_3_ (Supplementary Fig. S7). However, a significant decrease in the Mg:Ca ratio was observed at the highest concentrations of matrix (Fig. 4), suggesting that the presence of matrix inhibits the incorporation of Mg into the resulting mineral. Interestingly, *in vivo* produced intestinal precipitates contained 24.4 ± 1.1% MgCO_3_ (mean ± SEM), which lies within the range (~6–35%) of the values observed in the *in vitro* assay.

## Discussion

Results from the precipitate purifications and subsequent MS analyses clearly demonstrate the presence of an organic matrix associated with the intestinal precipitates produced by the Gulf toadfish. To our knowledge, this is the first time such a matrix has been described in this biomineralization system, and provides the first evidence for how the precipitation reaction that results in intestinal carbonate formation is directly regulated at the molecular level. It has been well demonstrated that intestinal bicarbonate and calcium concentrations, the substrates required for CaCO_3_ formation, are controlled via various mechanisms, which can affect the rate of CaCO_3_ formation. Regulators of intestinal bicarbonate include the guanylin peptides^31^ and prolactin^32^, where calciotropic hormones such as parathyroid hormone-related protein (PTHrP) and stanniocalcin (STC) can modify both luminal bicarbonate and calcium concentrations^33^,^34^. Additionally, the presence of calcium itself can modify the rate of intestinal bicarbonate secretion^35^. Although all of these regulatory molecules can vary the ion composition of the intestinal lumen, which can in turn modify the rate of CaCO_3_ production, the presence of an organic matrix in the precipitates provides a point at which the precipitation reaction can be controlled directly, allowing for more precise control of this important osmoregulatory process.

It is worth noting that although the presence of keratins and trypsin in MS samples is often indicative of sample contamination, these proteins are expected in these analyses due to their high abundance in the intestine^36^,^37^. Although some contamination by human keratin and trypsin autolysis is unavoidable, the inclusion of the cRAP proteins in the search database should prevent the accidental identification of fish proteins from human protein contaminants. Further, a subset of the identified peptides assigned to the toadfish keratins and trypsin were compared to the human and porcine sequences, respectively, and they were not identical. Therefore, we believe that the keratins and trypsin identified were indeed derived from the samples, and are not contaminants.

Acidic proteins were identified in samples purified by both methods, with 15 and 8 proteins in the gentle and stringently purified matrix, respectively, having an isoelectric point (pI) less than five. This relatively large percentage (~10% in the gentle samples and ~17% in the stringent) of the identified proteins with acidic properties is a common characteristic of biomineralization matrices^13^. The acidic nature of these proteins, along with the presence of known calcium-binding motifs, likely allows for their interaction with the cationic calcium ions. Another protein, carbonic anhydrase, which is often associated with carbonate containing minerals, was also observed in insoluble fraction of the gently purified precipitates. This enzyme catalyzes the interconversion of CO_2_ to HCO_3_^−^, and is known to be involved in controlling the precipitation reaction in numerous biomineralizing systems such as the coral skeleton and mollusk shell^38^,^39^. The presence of this protein near the site of calcification in the intestine could modulate that rate of CaCO_3_ production by controlling the local concentration of HCO_3_^−^. This along with the calcium binding properties of several of the proteins also found in the matrix (Supplementary Tables S2 and S3) presents potential mechanisms to control local concentrations of both Ca^2+^ and HCO_3_^−^; the two requirements for CaCO_3_ production.

The MS results from the two different purification methods allows for delineation between all the proteins that are associated with the precipitates (gentle purification), and only those which are found fully embedded within the CaCO_3_ mineral (stringent purification). The presence of large amounts of actin and actin-binding proteins (plastin-1, villin-1, etc.) as well as myosin-9 in the gently purified precipitates, but lower abundance in the stringently purified samples, suggests that these cytoskeletal proteins may be involved in forming or maintaining the ultrastructure observed in the precipitates formed *in vivo*. The presence of four different annexins in the gently purified matrix is also extremely interesting. Annexins are calcium-binding proteins that are highly abundant in the matrix vesicles responsible for mineralization of bone. During bone formation, these intracellular matrix vesicles serve to concentrate calcium and phosphate so amorphous calcium phosphate spontaneously precipitates^40^. It is believed that these vesicles are then exocytosed into the extracellular matrix, where the mineral is deposited on the extracellular matrix, forming the mature hydroxyapatite^41^. It has been shown that annexin A2, annexin A5 and annexin A6 are found only in vesicles where mineralization occurs, and that these three proteins form Ca^2+^ channels that serve to concentrate calcium in the vesicles^40^. With the identification of two (annexins A2 and A5) out of the three of these proteins in loose association with the intestinal precipitates, one could hypothesize that a similar process occurs in the toadfish intestine, and that at least some part of the precipitation occurs within a modified vesicle. The presence of carbonic anhydrase (discussed above) in these vesicles would additionally allow for the regulation of internal bicarbonate concentrations, adding another dimension of control over the precipitation reaction. Clearly, this hypothesis requires further investigation.

It is intriguing that a large number of the proteins identified in the matrix samples are known to reside in different subcellular components, including the endoplasmic reticulum, cytoplasm as well as the plasma membrane. Although it would be expected that the majority of the proteins found in the intestinal fluid, and hence the intestinal precipitates, would be secreted proteins, the high rates of enterocyte turn-over and cell shedding may explain the relatively high abundance of these proteins in the precipitates and the intestinal fluid^42^,^43^. Further, the exact location of the beginning of the precipitation reaction has not been investigated in the teleost intestine. As discussed above, it is possible that the precipitation reaction begins in some sort of microenvironment, such as a modified cellular vesicle similar to the matrix vesicles that are involved in bone formation^44^. Precipitation in cellular vesicles could also explain the presence of non-secreted proteins in the precipitate matrix.

Further, the clear enrichment of several proteins in the organic matrix as compared to the intestinal fluid suggests that the presence of the proteins is not solely due to non-specific contamination from proteins present in the intestinal lumen. It is intriguing that the second most abundant protein in the stringently purified precipitates, NADPH-cytochrome P450 reductase, is not observed in the intestinal fluid. This protein is usually found in the endoplasmic reticulum and serves an electron donor to several different oxygenase enzymes, which are involved in the oxidative metabolism of both endogenous, and exogenous compounds^45^. Another protein, phospholipase A2, was one of the most abundant proteins in the stringently purified precipitate matrix, but was also not identified in the intestinal fluid. Although no Ca^2+^-binding sites were identified in this protein, homologous proteins in other species are well known for their ability to bind calcium^46^, a common characteristic of proteins involved in the regulation of biomineralization systems where calcium-containing minerals are produced^17^. It is unknown what role, if any, these proteins may play in the intestinal precipitate matrix, but it certainly presents an interesting subject for future investigation.

There are multiple possible explanations for why several proteins were identified only in the precipitate organic matrix, and not in the intestinal fluid. It is first worth noting that although mass spectrometry is a highly sensitive technique, the analysis of the intestinal fluid proteome performed here is undoubtedly incomplete due to the complexity of the samples. Therefore, proteins with low abundance may not have been identified in these analyses. More in-depth investigations of the intestinal fluid would require fractionation of the samples prior to analysis, and longer analysis times. These experiments are currently ongoing. Additionally, it is possible that some proteins are indeed only in the precipitate matrix, and not the intestinal fluid. Should this be the case, it would provide further evidence that vesicle-type structures are involved in the formation of the precipitates, as that would allow for the incorporation of proteins into the matrix that are not present in the intestinal fluid. Further investigation of these topics is clearly needed.

Interestingly, three proteins (truncated accession numbers of *de novo* transcripts: Muscle_L_6293_T_1/4, Muscle_L_12311_T_1/1, and Muscle_L_233_T_1/3) were identified in the stringently purified precipitate matrix that did not show substantial homology to any proteins with known function. Even more intriguing is that two of these (Muscle_L_12311_T_1/1 and Muscle_L_233_T_1/3) were identified only in the precipitate matrix and not in the intestinal fluid. The use of the LocTree2 framework embedded in the PredictProtein tool suggests that both of the proteins are likely secreted^47^,^48^. Additionally, these proteins are suspected to have large areas of intrinsic disorder^49^, which is characteristic of many proteins involved in biomineralization^13^,^18^. Further, despite having an only slightly acidic pI (6.75), the protein encoded by transcript Muscle_L_233_T_1/3 has several clearly acidic domains, including one glutamic acid rich, 39 amino acid region with a pI under four. Detailed investigation of these proteins and their potential role in the control of CaCO_3_ precipitation is warranted.

The *in vitro* calcification assay results indicate that the stringently isolated precipitate matrix proteins are capable of modulating the precipitation reaction in a highly dose-dependent manner, with low concentrations of matrix proteins stimulating CaCO_3_ precipitation, and high concentrations having an inhibitory effect. Similar trends have been reportedly previously with both synthetically produced short peptides^50^, as well as water-soluble proteins extracted from the nacre of the sea snail (*Haliotis laevigata*)^51^. For intestinal precipitation in teleosts, the implications of the opposite effects on calcification at the extreme concentrations are intriguing, as theoretically the calcification reaction in the intestine could be tightly regulated without changes in gene transcription or protein translation. When the fish imbibes water, the proteins in the intestine would become more dilute, increasing the rate of CaCO_3_ production and aiding water absorption across the intestinal epithelium. This absorption would serve to concentrate the remaining proteins in the intestinal lumen, which would halt further precipitation that could potentially cause an intestinal blockage. Intriguingly, an estimated 85% of imbibed seawater is absorbed across the teleost intestinal epithelium^4^. Assuming the absolute quantity of matrix protein remains constant in the intestinal fluid, the concentration of these proteins would change by approximately seven-fold between the time the water was imbibed, and the completion of water absorption by the intestine. According to our calcification assay results (Fig. 3), this change in concentration would be sufficient to substantially increase the rate of calcification immediately post-ingestion, and inhibit further CaCO_3_ production after water absorption is completed. Currently, further testing of this hypothesis is difficult due to the challenges associated with determining the concentration of matrix proteins *in vivo*. From our experience, total intestinal protein concentrations in fasted toadfish range from ~500 to 1200 μg/ml (personal observation), which is over three orders of magnitude higher than the matrix concentrations tested here. However, clearly only a small fraction of the proteins in the intestinal lumen are biomineralization related, and we see no immediate way to quantify the concentration of this subset of proteins. In future research, once individual proteins of interest can be determined, targeted experiments looking at the concentrations of these proteins in the intestinal lumen should be completed.

The mechanism behind the observed enhancement of precipitation with low concentrations of matrix, and inhibition at high concentrations is unknown. Previous work with synthetically produced peptides found that individual peptides are capable of similar trends to those observed here, with stimulation and inhibition of CaCO_3_ production at low and high concentrations, respectively^51^. The authors of this previous study suggest that the enhancement of precipitation at low concentrations may be due to a decrease in the magnitude of the diffusive barrier and subsequent decrease in the energy barrier for attachment of solutes to the already formed CaCO_3_ due to the presence of the peptides. Interestingly, this effect correlated with both the hydrophilicty and net charge of the peptides. The mechanism of inhibition observed with the precipitate matrix is likely due to the well characterized binding of the proteins to crystal step edges, preventing the further growth of crystal layers^52^,^53^. Whether the observed effects of the isolated precipitate matrix on precipitation are due to a small subset of the proteins present in the matrix, or the interaction of many proteins remains unknown. Fractionation of the precipitate matrix and analysis of these fractions would serve to address this question, and is currently ongoing.

The pH-stat experiments used to validate the results from the newly designed micro-modified calcification assay showed similar trends for nucleation time to those observed in the calcification assay. However, no significant changes in max calcification rate were observed regardless of the concentration of organic matrix, although a trend toward increased max calcification rate was observed at the lowest concentration tested (0.05 μg/ml) compared to the no protein control. We hypothesize that this lack of change in the max calcification rate was an artifact of the high rates of mixing required for proper operation of the pH-stat titration system, and argue that the conditions in the micro-modified calcification assay with intermittent mixing are more representative of the conditions found *in vivo*. This fast rate of mixing, or the maintained high pH, also likely explains the substantial decrease in the observed time scale for calcification in the calcification assay (on the order of hours; Supplementary Fig. S3) as compared to the pH-stat experiments (on the order of minutes; Supplementary Fig. S5). An apparent increase in the potency of the matrix was also observed in the pH-stat experiment when compared to the calcification assay, with lower protein concentrations exhibiting effects in the pH-stat assay that were only observed at higher concentrations in the calcification assay. This could also be due to the increased mixing rate, variability between isolated matrix samples, or the high pH maintained throughout the experiment, but the exact cause is unknown.

Although efforts were taken to ensure that the proteins in the stringently purified matrix samples that were used in the calcification assay and pH-stat experiments remained in their native, non-denatured state, the required processing steps may have had an effect on their conformation. It seems unlikely however, that any denaturation that may have occurred would enhance the observed control over calcification. Instead, it seems that protein denaturation would actually diminish the observed effects. To test this idea, future studies should express and purify a subset of the matrix proteins identified in the MS analysis, and look at their individual effects on the control of CaCO_3_ precipitation. Investigation into the presence of other macromolecules (such as carbohydrates and lipids) in the matrix would also be interesting.

Results from the EDS analysis of the CaCO_3_ produced *in vitro* in the presence of stringently purified intestinal precipitate matrix shows that the matrix proteins are capable of modulating the Mg incorporation into the mineral. This study is not the first to show such an effect in a biomineralization system. A simple hydrophilic peptide, as well soluble organic matrix extracted from the tests and spines of the sea urchin, *Paracentrotus lividus*, have both been shown to modify the incorporation of Mg into *in vitro* produced CaCO_3_ in a dose-dependent manner^54^,^55^. Interestingly, in both of these studies, an increase in the concentration of the additive lead to an increase in the Mg incorporation, opposite of the trend observed here with the isolated intestinal precipitate matrix. This could be related to the relatively unique ion composition of the intestinal lumen, where Mg^2+^ concentrations can exceed 100 mM^5^.

The possibility that the organic matrix found in the intestinal precipitates may be at least partially responsible for determining the Mg content of the formed precipitates is intriguing due to the large variations in Mg content observed in intestinal precipitates produced across species^10^. Different species have been shown to produce high magnesium calcite, ranging from <1% to >60% MgCO_3_^29^. It is unlikely that these large differences in Mg concentration are a consequence of variation in the inorganic chemistry at the site of precipitation, as the Mg^2+^ to Ca^2+^ ratio in the intestinal fluid is set almost entirely by the imbibed seawater due to the relatively impermeable nature of the gastrointestinal tract to these ions^4^,^12^. The aforementioned experiments suggest that changes in precipitate matrix concentration, and likely composition as well, may explain the interspecific differences in the Mg content of the intestinal derived precipitates. The effects of the organic matrix on the mineralogy of the precipitates requires substantially more investigation, but changes in Mg incorporation observed here suggests that it may play a role in determining the structure of the resulting mineral.

The results presented here are the first to describe the molecular mechanisms behind an essentially uncharacterized biomineralization system: CaCO_3_ production in the marine teleost intestine. With ocean pH consistently decreasing due to the increased anthropogenic production of CO_2_^56^, the importance of understanding CaCO_3_ production in the ocean is more crucial now than ever, due to the impacts of these processes on ocean chemistry and the ability of the ocean to sequester atmospheric CO_2_. Recent studies have suggested that high Mg-calcites, such as those produced in the marine teleost intestine, might serve as “first responders” to ocean acidification due to their high solubility compared to other carbonate minerals^11^. With conservative estimates suggesting that 3–15% of the total carbonate production in the oceans forms in the marine fish intestine^6^, it is crucial that we understand the mechanisms that regulate this process. MS analyses completed here revealed more than 150 proteins associated with the precipitates produced in the intestine of the Gulf toadfish, and the use of a novel, micro-modified calcification assay showed that these proteins combined modified the rate of calcification in a dose-dependent manner. This suggests that the precipitation of CaCO_3_ in the marine fish intestine is tightly regulated by an organic matrix associated with the forming mineral.

## Methods

### Experimental animals

Gulf toadfish (*Opsanus beta*) were collected by shrimp fisherman operating out of Dinner Key, Miami, FL, and held in 60 L aquaria at the Rosenstiel School of Marine and Atmospheric Science on flow-through, filtered sea water from Biscayne Bay (32–37 ppt, 21–26 °C). Immediately upon arrival to the lab, fish were treated for ectoparasites with malachite green and a one-minute freshwater dip^57^. Fish were fed to satiation once weekly with squid, but were fasted for a minimum of seven days prior to experimentation to ensure the intestine was completely void of undigested material. All experimental methods were completed in accordance with relevant guidelines and regulations, and all experimental protocols were approved by the University of Miami Animal Care and Use Committee (protocol no. 13-225), which is accredited by the Association for Assessment and Accreditation of Laboratory Animal Care (AAALAC).

### Extraction and purification of intestinal precipitates: gentle purification

Twenty-four toadfish (two replicates of 12 fish each) were randomly selected from holding tanks and euthanized by an overdose of tricaine methanesulfonate (MS-222) and subsequent cervical dislocation. The sample size was determined by preliminary experiments, which revealed that precipitates needed to be collected from 12 fish and combined in order to obtain the 10 μg of protein necessary for subsequent mass spectrometry analysis. The intestine was then exposed by a ventral, midline incision, and promptly clamped at the pyloric sphincter, and immediately anterior to the rectal opening. The intestine was then dissected from the fish, the posterior clamp was removed, and the intestinal contents were gently squeezed into a microcentrifuge tube. Solid material (including the precipitates) were rinsed 4x in ultrapure water and then incubated 2x in 1 ml of 8 M urea and 1% SDS at 95 °C for 20 minutes. The urea/SDS solution was then removed, and the solids were incubated for 10 min in subsequent 1 ml, acetone, ethanol, and water washes. The dense CaCO_3_ mineral was then separated from remaining contaminants by glycerol gradient centrifugation. Glycerol gradients consisted of 300 μl layers of 100%, 85% and 75% glycerol in a 1.7 ml microcentrifuge tube. The samples were placed directly on top of the 75% glycerol layer and then centrifuged at 2,000 g for 2 min. The glycerol was then decanted, leaving the pellet consisting of the dense CaCO_3_ in the tube. The pellet was washed 2x in ultrapure and then dried in a vacuum evaporator for 30 min.

### Collection and purification of intestinal precipitates: stringent purification

A maximum of ten toadfish were randomly selected and transferred to 25 L aquaria filled with 1 μm filtered, UV-sterilized seawater (32–37 ppt). Fish were kept in the experimental tanks for no more than two weeks. Every 24 hours, tanks were siphoned and precipitates were collected by filtering the extracted water through fine (~500 μm) mesh. After siphoning, tanks were refilled with sterile filtered seawater, resulting in a minimum 60% water change. Collected precipitates were rinsed 2x in ultrapure water and then incubated in a NaOCl solution containing 5% available chlorine for 24 h at 4 °C. The purified CaCO_3_ was then collected by centrifugation for 3 min at 2,000 g and the resulting pellet was rinsed 2x in ultrapure water. Samples collected over multiple days from the same tanks were pooled in order to obtain the required amount of sample.

### Extraction of precipitate organic matrix and collection of intestinal fluid samples

EDTA was chosen to remove the inorganic mineral as opposed to dilute acid due to the small quantities of precipitates collected from each fish. Preliminary experiments found that adding the required amount of acid to dissolve the mineral, without making the solution overly acidic (which could degrade the proteins) was difficult. Therefore, CaCO_3_ was removed from purified precipitates by the addition of excess 0.5 M EDTA (pH 8.0), and incubation at 4 °C for 3 h. The resulting solution was centrifuged at 2,000 g for 10 min. Both the pellet and supernatant were collected as the EDTA insoluble and EDTA soluble fractions, respectively, except for in the stringently purified samples, where only soluble samples were collected as no substantial pellet was observed. The insoluble fractions were rinsed briefly 3x in ultrapure water to remove residual EDTA and resolubilized in 8 M urea. The supernatant from multiple samples were combined as necessary, diluted 3-fold with ultrapure water, and then concentrated using centrifugal filter units (3 kDa molecular weight cutoff). The buffer in the concentrated samples was exchanged into tris buffered saline (TBS; Supplementary Table S4) by three, minimum 8-fold dilutions with TBS and subsequent concentration in the centrifugal filter units. After the final buffer exchange, the sample was concentrated to a volume of 200 μl and dialyzed (3.5 kDa molecular weight cutoff) into TBS overnight to ensure the complete removal of EDTA. Intestinal contents for the intestinal fluid samples were collected using the extraction procedure described for the gently purified precipitates. Contents were centrifuged briefly after extraction and only the supernatant was retained as the intestinal fluid sample. Protein concentrations for both the extracted matrix and the intestinal fluid were determined by the bicinchoninic acid (BCA) method^58^. Samples were stored at −80 °C until analysis.

### Mass spectrometry

All mass spectrometry analysis was completed at the Colorado State University Proteomics and Metabolomics Facility (Fort Collins, Colorado). Purified proteins were reduced, alkylated, and trypsin digested as described previously^59^. Digested peptides were reverse phase fractionated using a 90 min linear gradient from 10–30% acetonitrile and eluted into an Orbitrap Velos Pro (Thermo Scientific) mass spectrometer operating in data dependent acquisition mode. A full description of the instrumentation and acquisition settings used can be found elsewhere^60^. Raw data from the MS was searched against the *O. beta* transcriptome using the Mascot database search engine (v2.2, Matrix Science). Prior to searching, nucleotide sequences generated from all four groups of tissues (Supplementary Table S1) were combined, and translated into all six possible open reading frames. Sequences that were at least 90% similar were then removed using CD-HIT^61^, which resulted in a database containing ~346 k sequences. Sequences of common laboratory contaminants from the common Repository of Adventitious Proteins (cRAP; http://www.thegpm.org/crap/) were then added and a reverse concatenated database was produced using Scaffold (v4.4, Proteome Software). Searched data was loaded into Scaffold where protein and peptide cut-off thresholds were set at 99.0% and 95%, respectively, and a minimum of two unique peptides were required for protein identification. Proteins observed in the isolated matrix with only two unique identified peptides were manually validated to ensure the spectra were of high quality. Probable protein identifications were determined by a combination of the BLAST2GO pipeline^62^, and manual BLAST searches against the NCBI non-redundant database. Despite the removal of duplicate sequences from the MS search database prior to searching, BLAST analysis revealed that several of the gene products with different accession numbers identified in the MS analyses were from the same protein. Therefore, these proteins were grouped in Supplementary Tables S2 and S3, and elsewhere, but all accession numbers are listed individually. All mass spectrometry data were deposited to the ProteomeXchange Consortium via the PRIDE^63^ partner repository under the identifiers PXD004134 and PXD004135.

### Micro-modified calcification assay

A micro-modified *in vitro* calcification assay was developed to measure the rate of CaCO_3_ formation in the presence of isolated precipitate matrix. Instead of measuring the rate of CaCO_3_ production directly, this method monitors the production of H^+^ ions as a proxy for calcification:


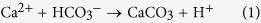


The pH of a solution containing Ca^2+^, HCO_3_^−^, and the sample of interest is monitored over time, and the rate of pH drop is used to estimate the rate of CaCO_3_ production. These so-called ‘pH drift assays’ have been well utilized previously^64^,^65^, but they typically require the reaction to be done in relatively large volumes (at least several ml) in order to accommodate the electrode required for pH measurements. Here, thymol blue, a pH indicator which shows a color change from approximately pH 7.6–9.0, was added to the solution, which allowed for pH to be monitored by absorbance at 594 nm. This allowed the reactions to be completed in 96-well microplates, decreasing the required volume for the reaction to 200 μl (and therefore also decreasing the amount of sample required to achieve the desired protein concentrations), and increasing the throughput, allowing up to 88 samples to be analyzed simultaneously along with the required standard curve. An example of the raw data collected from this micro-modified calcification assay is shown in Supplementary Fig. S3.

Stringently purified organic matrix samples were diluted with TBS as necessary to produce protein concentrations that were 10-fold more concentrated than the desired final concentration in the assay. Gently purified matrix was not tested here, as the purification procedure likely denatured the proteins, making the results unreliable. A pH standard curve ranging from 7.6 to 9.0 by increments of 0.2 pH units was produced in the buffer detailed in Supplementary Table S4. A 200 μl aliquot of each of these standards was then added to a 96-well microplate in triplicate. The remainder of the wells was filled with 90 μl of the bicarbonate buffer, 20 μl of the diluted matrix samples (in TBS), and finally 90 μl of calcium buffer (Supplementary Table S4). The 1 mM tris that remained in the wells after the dilution of the sample in TBS by the other reagents still allowed for sufficient changes in pH to be detected throughout the assay, and likely had little effect on the precipitation reaction itself. After addition of the calcium buffer, the plate was immediately sealed with an optically clear plate sealing film and inserted into a SpectraMax Plus 384 (Molecular Devices) microplate reader. The optical absorbance at 594 nm was read every 30 s for 30 min, and then once every 5 min for 19.5 h. Prior to each reading, the plate was shaken for 3 s. The temperature of the plate was maintained at 26 ± 1 °C throughout the duration of the experiment. Absorbance data was recorded in SoftMax Pro (v6.4; Molecular Devices).

Upon completion of the assay, a pH standard curve was produced by fitting a first order polynomial equation to the average 594 nm absorbance of the pH standards throughout the assay. Raw absorbance values for the samples were then transformed to pH using this curve. Control experiments where identical samples were analyzed in the presence and absence of thymol blue revealed that the absorbance of light at 594 nm by the CaCO_3_ that forms during the assay was minor, and had little effect on the calculated pH values, or the results of the assay (Supplementary Fig. S8). Although traditionally the drop in pH is directly used to monitor the rate of CaCO_3_ production^17^,^65^,^66^, we chose to instead transform pH values into H^+^ concentration, in order to remove any influences of the logarithmic nature of the pH scale from further quantification. Nucleation time for each sample was calculated by determining the time at which the H^+^ concentration in each well had increased by 10% of the total increase observed throughout the 20 h experiment (Supplementary Fig. S3). Calcification rate was calculated by taking the slope of a linear regression fitted through the data from 20–60% of the total H^+^ concentration increase observed. The calculated nucleation times and calcification rates were then normalized to a no protein control (a sample that had undergone all of the purification procedures completed in the matrix samples, but did not contain any protein), as preliminary experiments found that this normalization regime reduced variability between assays. Note that the transformation from pH to H^+^ concentration had little effect on any of the aforementioned calculations once values were normalized to the no protein control (Supplementary Fig. S3). Although a shift in the absolute nucleation times can be observed in Supplementary Fig. S3 when H^+^ concentration is used instead of pH, this difference is abolished post-normalization, suggesting the difference is due to a systematic shift among all of the samples between the calculation methods. Since all values are normalized to the control prior to further analysis, this shift does not affect the conclusions. To quantify the effects of matrix, normalized nucleation times and calcification rates were compared to the respective values for the no protein control by one-way ANOVA and subsequent multiple comparisons using the Holm-Sidak method. All replicates were collected from different fish and are therefore biological replicates. Prior to statistical analysis, all values were normalized to the respective no protein controls. In addition, the nucleation times were log transformed, and a constant of 1.0 was added to the calcification rates, which were then also log transformed to obtain equal variances (as determined by the Brown-Forsythe test).

### Scanning electron microscopy and energy dispersive spectroscopy

The same buffers that were used in pH-stat experiments were used for the SEM and EDS experiments. Bicarbonate buffer (90 μl), matrix proteins in TBS (20 μl), and calcium buffer (90 μl) were mixed in a microcentrifuge tube and immediately transferred to the top of an aluminum specimen mount. Stubs were then placed in a sealed box that was partially filled with water in order to provide a humid environment for the precipitation reaction to occur. The sealed box was placed in a 26 °C incubator for 20 hours, after which the stubs were removed and the fluid on the stubs was decanted. This left behind the CaCO_3_ that formed in direct contact with the stub. Excess salt was removed from the mineral by three brief washes with ultrapure water, and then the stubs were dried at 37 °C for 1 h. After drying, stubs were coated in palladium and analyzed on an FEI XL-30 field emission ESEM/SEM, fitted with an Oxford EDS system. All EDS analyses were completed at 300x magnification, and data were collected until the Ca^2+^ peak reached 1000 counts. Three EDS readings were obtained at random positions within each sample and averaged. All values were compared to no protein controls via one-way ANOVA and Holm-Sidak multiple comparisons.

## Additional Information

**How to cite this article**: Schauer, K. L. *et al*. A proteinaceous organic matrix regulates carbonate mineral production in the marine teleost intestine. *Sci. Rep.* **6**, 34494; doi: 10.1038/srep34494 (2016).

## Supplementary Material

Supplementary Information
