# Corrigendum: A proteinaceous organic matrix regulates carbonate mineral production in the marine teleost intestine

**DOI:** 10.1038/srep46844

**Published:** 2017-06-19

**Authors:** Kevin L. Schauer, Christophe M. R. LeMoine, Adrian Pelin, Nicolas Corradi, M. Danielle McDonald, Wesley C. Warren, Martin Grosell

Scientific Reports 6: Article number: 3449410.1038/srep34494; published online: 10 03 2016; updated: 06 19 2017

M. Danielle McDonald was omitted from the author list in the original version of this Article. This has been corrected in the PDF and HTML versions of the Article, as well as the Supplementary Information file that accompanies the Article.

The Acknowledgments section now reads:

“The authors wish to thank the staff at the Colorado State University Proteomics and Metabolomics Facility for their assistance with the mass spectrometry analyses presented here as well as Patricia Blackwelder at the University of Miami Center for Advanced Microscopy for her assistance with the electron microscopy. K.L.S. is supported in part by the University of Miami Maytag Fellowship. The transcriptome work was supported by NIH grant R24 RR032658-01 to W.C.W., McDonnell Genome Institute at Washington University School of Medicine. M.D.M. was supported by NSF (IOS-0920547). M.G. is a Maytag Chair of Ichthyology and is supported by NSF (IOS 1146695)”.

The author contribution statement now reads:

“K.L.S. and M.G. designed the experiments. K.L.S. performed the experiments, analyzed the data, and wrote the manuscript. M.G., M.D.M., W.C.W. and C.M.R.L. designed and executed transcriptomic experiments. W.C.W. performed the transcriptome sequencing. C.M.R.L., A.P. and N.C. analyzed, and assembled the transcriptomic data. M.G., C.M.R.L., A.P., W.C.W. and N.C. provided edits to a draft manuscript; M.G. approved the final version”.

