# Supplementary material for: A proteinaceous organic matrix regulates carbonate mineral production in the marine teleost intestine
Source: Sci Rep. 2016 Oct 3;6:34494. doi: 10.1038/srep34494 (PMC5046086; doi:10.1038/srep34494)
Supplement: Supplementary Information [file srep34494-s1.pdf]

Supplementary Information for:

A proteinaceous organic matrix regulates carbonate mineral production in the marine teleost intestine

Kevin L. Schauer, Christophe M. R. LeMoine, Adrian Pelin, Nicolas Corradi, Wesley C. Warren, and Martin Grosell

Supplementary Methods

Transcriptomics

Flash frozen tissues (Supplementary Table S1) were pulverised under liquid nitrogen, homogenized in Trizol (ThermoFisher Scientific) and RNA was extracted following the acid-phenol chloroform extraction protocol. The transcriptome of each category of tissues was generated by subjecting pooled RNA samples to Illumina sequencing at the Washington University Genome Institute (St Louis, MO). De novo transcriptome assembly was initiated using Velvet[1] (v1.2.07) for a k-mer value of 93 for all samples with parameters chosen by VelvetOptimiser (v2.2.0; http://www.vicbioinformatics.com/software.velvetoptimiser.shtml). For every sample, the assembly was processed by the Oases add-on as per author's instructions[2]. Assembled transcripts for all samples were individually imported into the Blast2GO pipeline[3] and annotated with GO terms as well as InterProScan domains[4]. Further, from each library we subtracted transcripts present in other libraries to generate a list of loci unique to each set of sequenced tissues. The GO term enrichment analysis of these unique libraries is represented as percent relative abundance per tissue set, computed from total count numbers for each category represented (Supplementary Fig. S2).

pH-stat experiments

Buffers identical to those used in the micro-modifed CA assay were used in the pH-stat experiments, but thymol blue was omitted. Bicarbonate buffer (675 µl) was added to a 2 ml,

water-jacketed vessel maintained at 25°C, followed by matrix samples diluted in TBS (150 μl), and finally 675 μl of calcium buffer. Samples were vigorously stirred throughout the duration of the experiment by a magnetic stir bar. The pH of the solution was measured by a PHC4000.8 electrode (Radiometer) attached to a TIM 854 or 856 automatic titrator, which delivered 0.2N NaOH (prepared from a standardized stock solution) via a microburette tip inserted into the solution. Titrant was added at the rate necessary to maintain a constant pH of 8.6. During the beginning of the experiment, NaOH was added rapidly in order to bring the pH up to 8.6, as the TBS which contained the matrix had a pH of 7.6. Once the pH of 8.6 was achieved by the initial base addition, the pH of the solution would slowly drift upwards until calcification began, which would in turn cause the pH to begin dropping. During the period of increasing pH, the microburette tip was removed from the solution in order to prevent it from being clogged by precipitation of $CaCO_3$. The pH of the solution as well as the addition of titrant was recorded every 20 s by a personal computer running Titramaster software (v5.1.0; Radiometer). The reaction was allowed to proceed until base addition had not occurred for a minimum of 5 minutes. Nucleation time was determined to be the time of max pH, due to the reasons described above (Supplementary Fig. S5). Max calcification rate was the max rate of titrant addition averaged over a 20s period (excluding the initial base addition to achieve a pH of 8.6; Supplementary Fig. S4). Nucleation times and calcification rates were normalized and compared to control experiments that contained no protein (TBS only) by one-way ANOVA and multiple comparison analyses by the Holm-Sidak method.

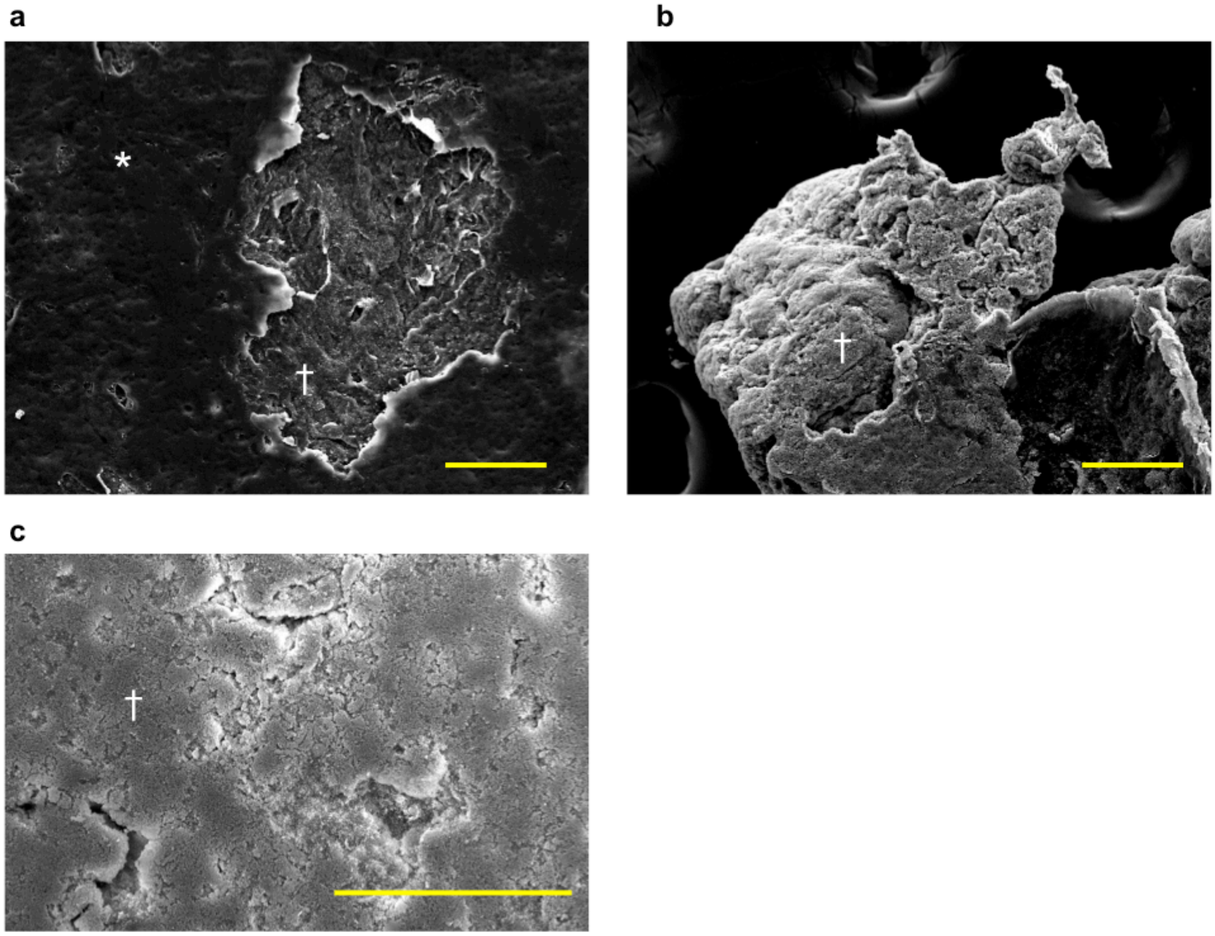

Supplementary Figure S1: Scanning electron microscopy (SEM) images of intestinal $CaCO_3$ precipitates. **(a)** Unpurified precipitates, **(b)** gentle purification procedure purified precipitates collected directly from the intestines of Gulf toadfish (*Opsanus beta*), and **(c)** precipitates collected from the tank bottom and purified using the stringent purification method. * Mucus coating; † exposed $CaCO_3$. Scale bar = 100 μm.

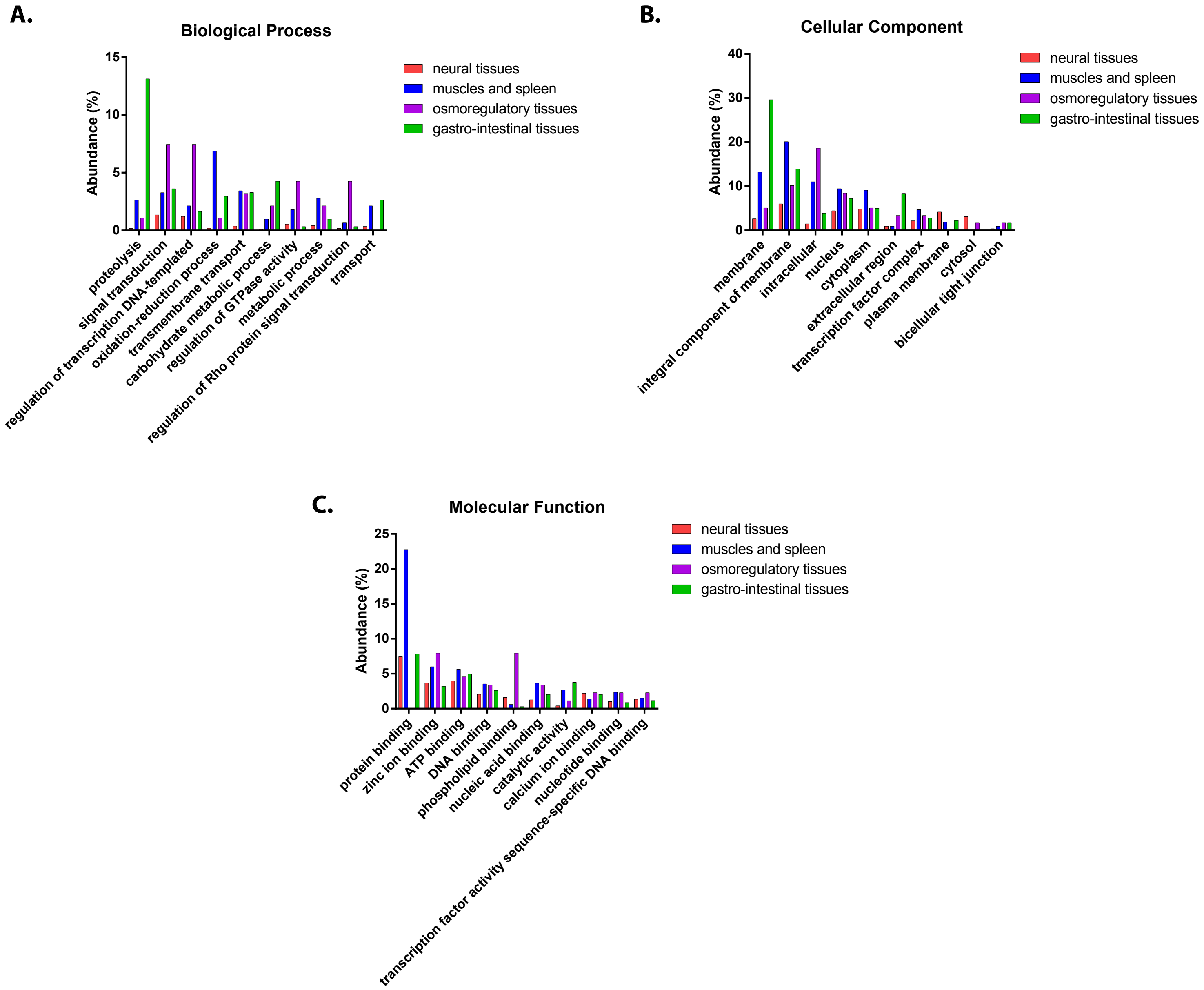

Supplemental Figure S2: Gene ontologies of unique transcripts in the toadfish functional transcriptome libraries. Unique transcripts within neural tissues (red), muscles (blue), osmoregulatory tissues (purple) and gastro-intestinal tissues (green) were annotated and gene ontology assigned in Blast2GO (see Methods). Within each library, the relative abundance of the most represented GO term was computed for **(A)** biological processes, **(B)** cellular components and **(C)** molecular function.

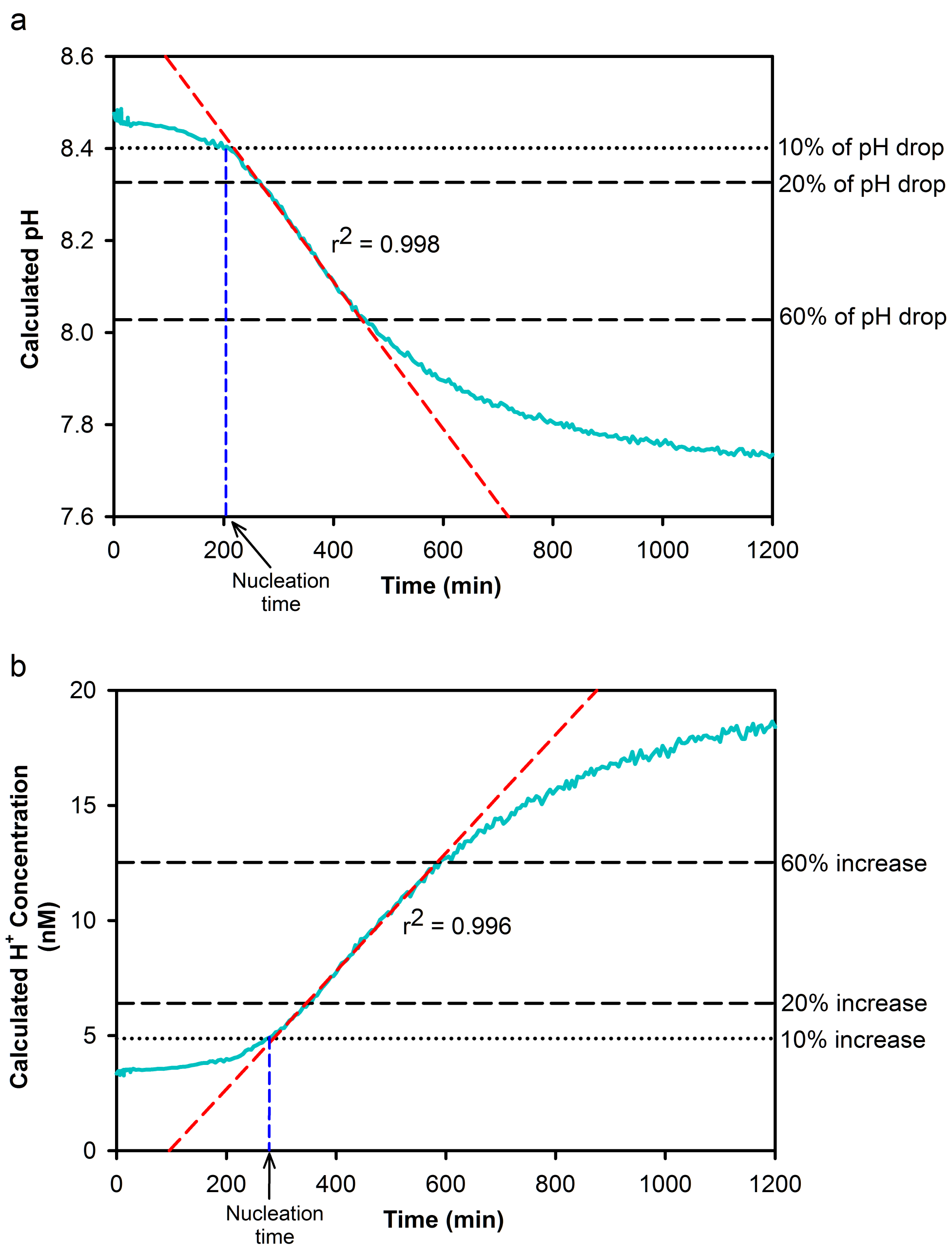

Supplementary Figure S3: Representative traces from the micro-modified *in vitro* calcification assay showing the comparison between pH **(a)** and H^+ concentration **(b)** and how nucleation time and calcification rate are calculated. Nucleation time (blue dashed line) is defined here as the time at which the pH or H^+ concentration has dropped or increased by 10% of the total change observed throughout the 20-hour assay. The calcification rate is calculated by taking the slope of a linear regression (red dashed line) fitted to the data between 20% and 60% of the total pH drop or H^+ concentration increase observed for the duration of the experiment. r^2 values for the linear regressions used to calculate calcification rate are also given, and show little difference between using pH of H^+ concentration.

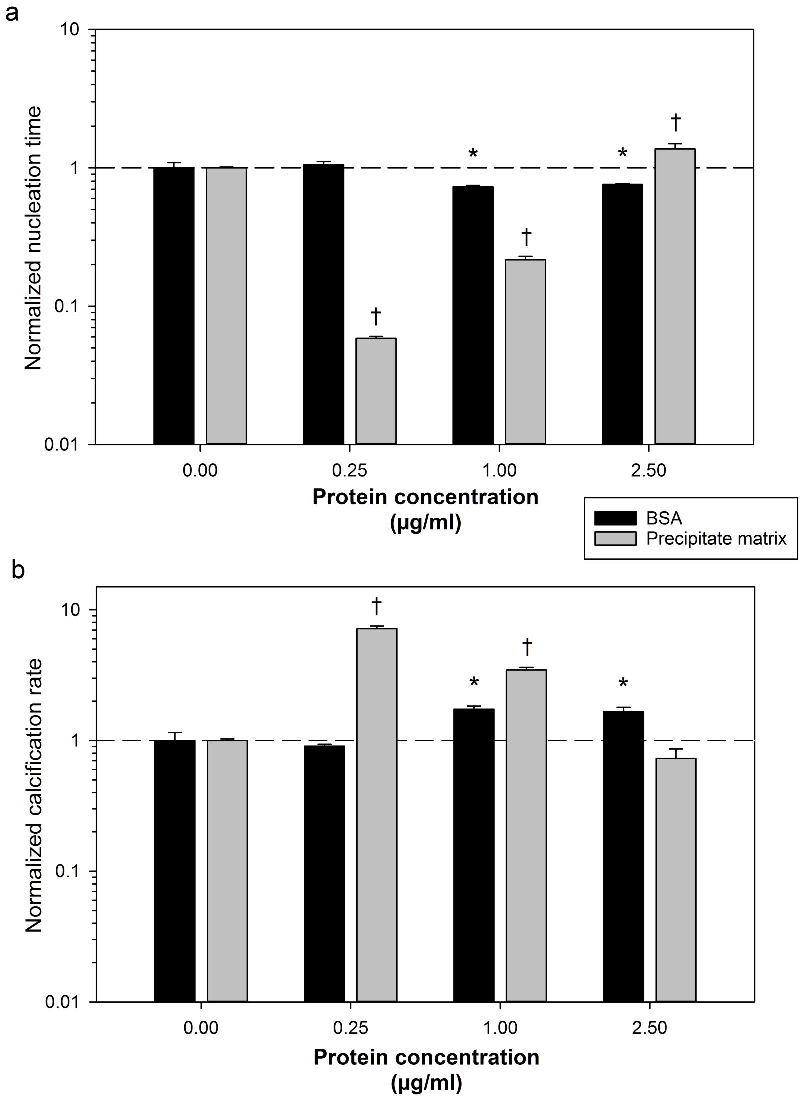

Supplementary Figure S4: The effect of BSA on *in vitro* $CaCO_3$ formation as compared to isolated precipitate organic matrix. Several concentrations of BSA were included in a calcification assay to determine if the effects observed in the presence of isolated organic matrix were due to non-specific protein interactions. Minor variations in both nucleation time **(a)** and calcification rate **(b)** were observed at the highest concentrations of BSA tested, but effects were minor compared to the precipitate matrix, suggesting the observed effects are not due to nonspecific protein interaction. Symbols (* BSA; † precipitate matrix) represent values that vary significantly ($p<0.05$) from controls, as determined by two-tailed ANOVA and Holm-Sidak multiple comparisons.

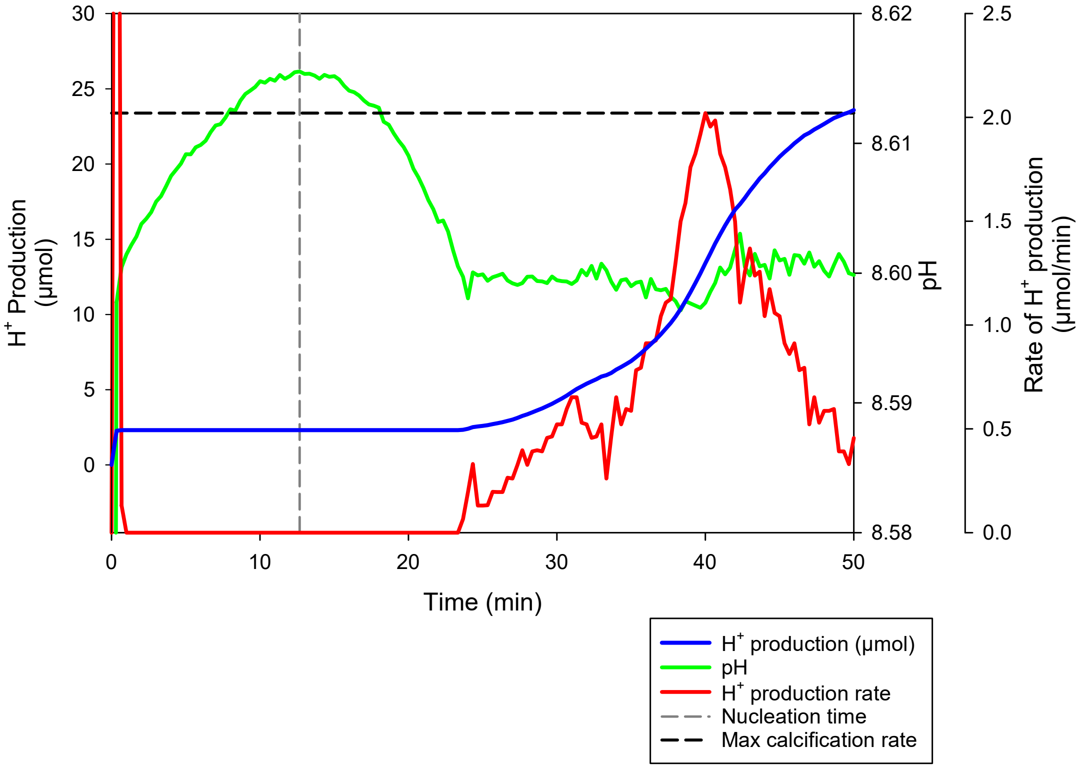

Supplementary Figure S5: Example trace from a single pH-stat experiment. The nucleation time (gray dashed line) was defined as the point of highest pH, as the outgassing of CO_2 causes a gradual increase in pH until the production of $CaCO_3$ begins. Max calcification rate (black dashed line) is the maximum rate of NaOH addition, which directly correlates to maximum rate of H^+ production, and therefore $CaCO_3$ production.

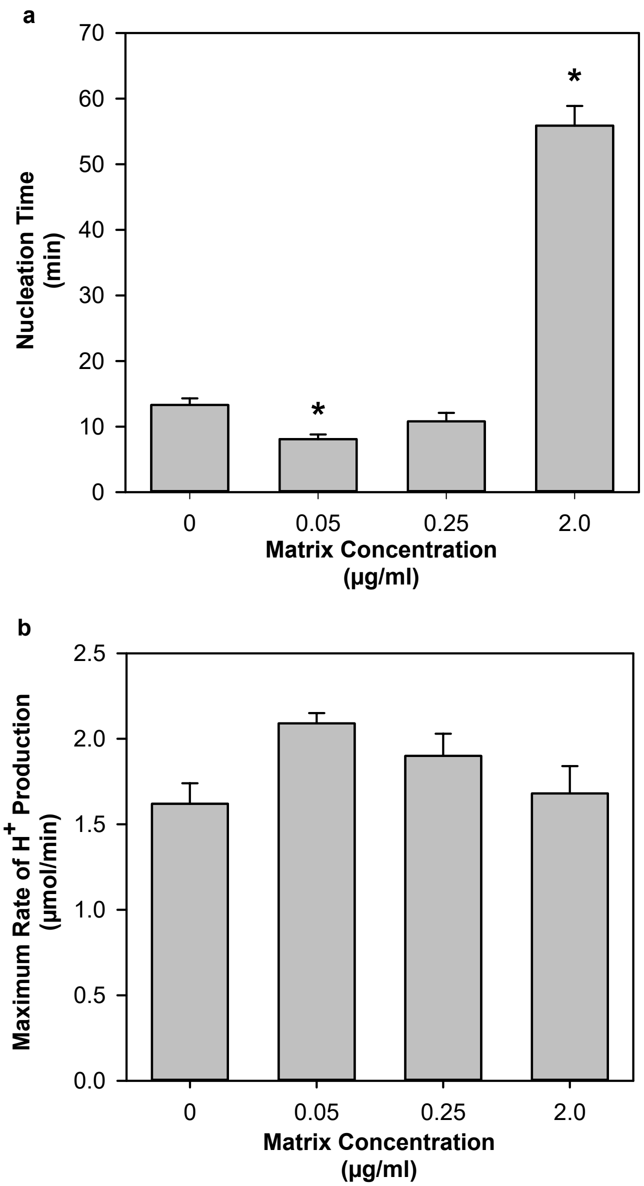

Supplementary Figure S6: Validation of micro-modified *in vitro* calcification assay using pH-stat. Purified intestinal $CaCO_3$ matrix were added to a pH-stat setup at several of the concentrations tested in the micro-modified calcification assay. **(a)** Nucleation time was defined as the point of max pH observed during the assay as the pH slowly drifts up prior to the start of calcification due to the outgassing of CO_2. **(b)** Max calcification rate was determined to be the max rate of NaOH addition as averaged over at 20-second interval. *$p \leq 0.05$ compared to no protein control as determined by ANOVA and subsequent multiple comparisons by the Holm-Sidak method. n=8 for no protein controls and n=5 for all other treatments.

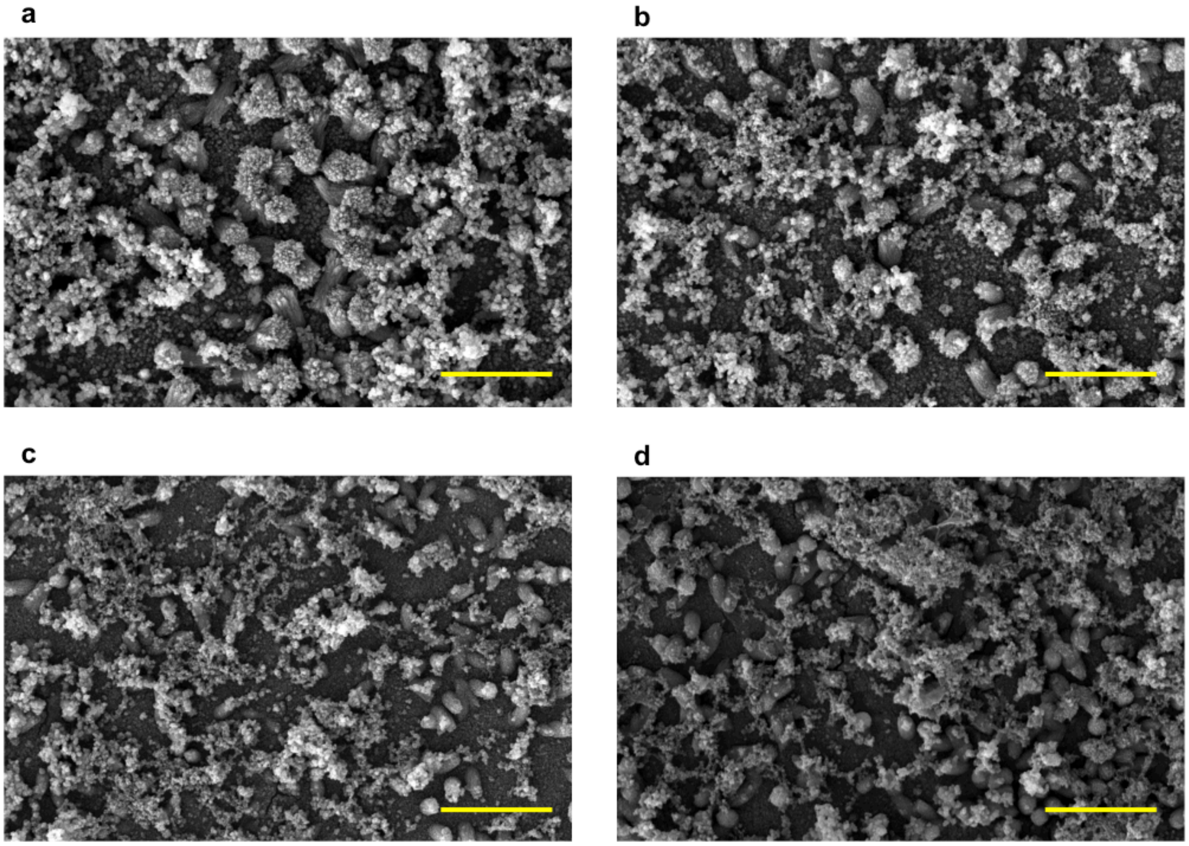

Supplementary Figure S7: Comparison of $CaCO_3$ crystal morphology formed *in vitro* in the presence of varying concentrations of purified intestinal precipitate matrix. **(a)** No protein, **(b)** 0.25 µg/ml matrix, **(c)** 2.0 µg/ml matrix, and **(d)** 5.0 µg/ml matrix. Scale bar = 20 µm.

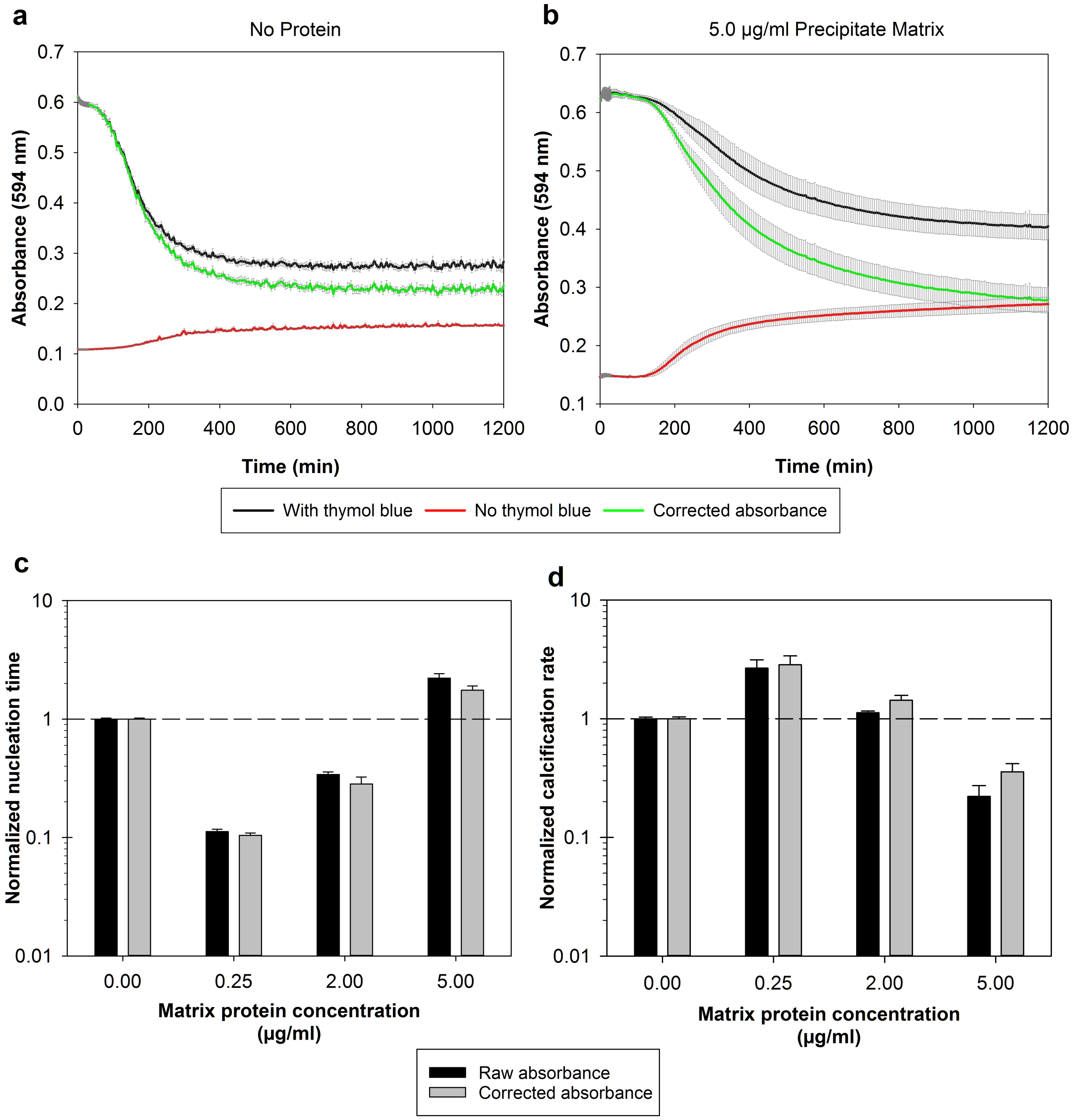

Supplementary Figure S8: The effect of the formation of $CaCO_3$ on the absorbance measurements used to calculate pH during the micro-modified calcification assay. Identical pairs of samples were analyzed in the assay, but in one sample thymol blue was omitted from the salines. This allowed for the measurement of the absorbance at 594 nm (the wavelength used to monitor pH) over time due to the formation of the $CaCO_3$ itself. The absorbance from the wells lacking thymol blue was subtracted from their paired samples that contained thymol blue, and both the raw absorbance and the corrected absorbance values were used to calculate the pH throughout the assay. Raw absorbance values for the wells with and without the thymol blue, as well as calculated corrected absorbances are shown for controls wells containing no protein **(a)**, as well as 5 µg/ml of isolated precipitate matrix **(b)**. Little change in nucleation time **(c)**, or calcification rate **(d)** was observed using the two absorbances, suggesting that the formation of $CaCO_3$ has a minimal effect on the outcomes of the assay. Values are shown as mean ± SEM. n=5-6.

a b

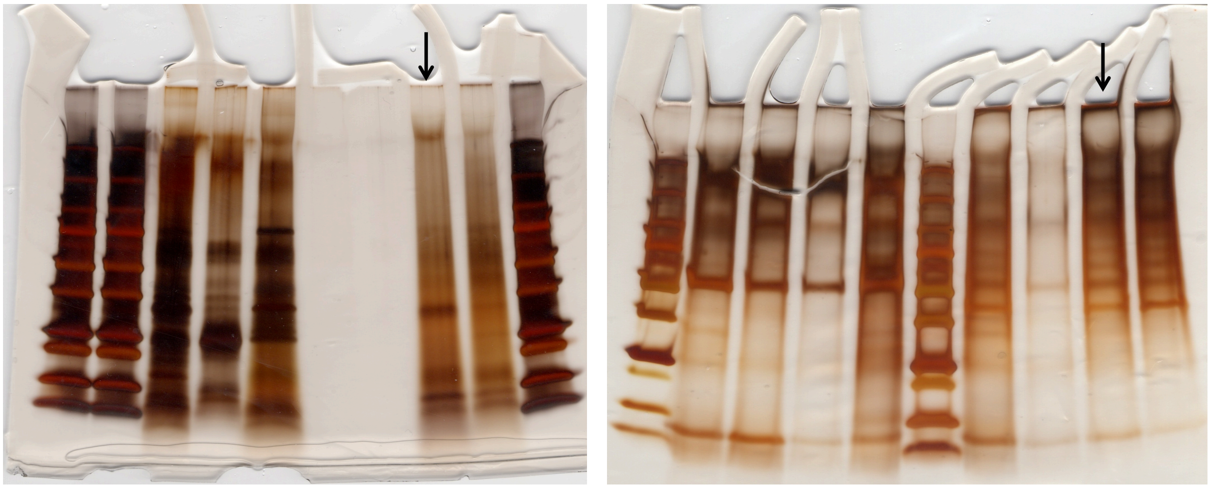

Supplementary Figure S9: Uncropped images of the gels shown in Figure 1. **(a)** The gel in which the EDTA soluble sample was run, and **(b)** the gel containing the EDTA insoluble sample. Lanes shown in Figure 1 are marked with an arrow. Other lanes represent samples from unrelated experiments and should be ignored.

Supplementary Table S1: Description of the assembled *O. beta* transcriptomes. The original tissues from which RNA was extracted and eventually sequenced is listed along with the NCBI identifiers for the assembled transcriptomes and raw sequence reads. All results can be found under BioProject PRJNA313355.

Transcriptome abbreviation	NCBI BioSample identifier	NCBI SRA identifier	Included tissues
Muscle	SAMN04520031	SRR3193034	Heart, mixed skeletal muscle, sonic muscle, spleen
GI	SAMN04520030	SRR3193033	Liver, intestine, stomach, Brockmann body, gallbladder
Osmo	SAMN04520029	SRR3192986	Kidney, gill, urinary bladder, skin
Neural	SAMN04520032	SRR3193035	Brain, eye, gas gland, gonad

Supplementary Table S2: Protein identifications by mass spectrometry in matrix extracted from precipitates purified using the gentle purification procedure. Biological replicates are listed separately and labeled numerically. Probable protein identifications were determined by BLAST of the NCBI non-redundant database. Proteins in gray are know contaminants from the cRAP database. Proteins in bold and italics contain known calcium binding domains. Accession numbers refer to *de novo* assembled transcripts and have been truncated for clarity.

Probable protein identification	Accession number	pI	Unique peptides				Total spectral counts			
			Insoluble		Soluble		Insoluble		Soluble	
			1	2	1	2	1	2	1	2
Trypsin (Porcine)	sp|TRYP_PIG|	7.22	6	6	7	9	193	138	265	484
Keratin, type II cytoskeletal 8-like	Neural_L_43_T_13/15	6.07	4	6	17	11	14	13	133	109
	Muscle_L_158_T_1/2	9.27	--	--	8	8	--	--	61	63
Keratin, type I cytoskeletal 10 (Human)	sp|K1C10_HUMAN|	4.87	11	11	26	18	24	20	116	59
Calmodulin	***Neural_L_113_T_4/5***	***8.85***	***--***	***--***	***6***	***7***	***--***	***--***	***89***	***91***
Keratin, type II cytoskeletal 1 (Human)	sp|K2C1_HUMAN|	8.34	10	9	23	19	29	24	123	95
Keratin, type II cytoskeletal 2 epidermal (Human)	sp|K22E_HUMAN|	8.17	6	8	25	20	11	16	82	64
Actin, cytoplasmic 2-like	Muscle_L_533_T_1/2	8.17	7	3	8	5	60	34	30	28
Keratin, type I cytoskeletal 9 (Human)	sp|K1C9_HUMAN|	4.95	8	4	17	16	18	7	55	51
Plastin-1	***GI_L_449_T_1/1***	***6.81***	***8***	***8***	***5***	***4***	***52***	***46***	***18***	***7***
Arginase-1	Muscle_L_144_T_13/14	10.15	3	3	4	3	61	41	16	13
Myosin-9	Muscle_L_3751_T_1/1	6.64	4	4	8	7	11	13	44	37
	Muscle_L_98_T_1/2	5.17	6	6	7	6	14	15	19	20
	Muscle_L_3751_T_1/1	6.64	2	--	--	--	7	--	--	--
	Muscle_L_4734_T_1/1	6.18	--	--	--	2	--	--	--	22
Keratin, type I cytoskeletal 13-like	Osmo_L_782_T_3/4	8.74	--	--	9	6	--	--	50	40
	Muscle_L_968_T_2/2	4.83	--	--	2	--	--	--	6	--
Na/H eschange regulatory cofactor NHE-RF3	GI_L_154_T_4/12	7.59	6	6	3	3	33	20	12	4
Tropomyosin alpha-4 chain	GI_L_360_T_5/8	6.55	2	2	6	5	4	7	24	25
	GI_L_744_T_1/1	4.85	3	3	4	3	9	10	8	10
Chitinase	GI_L_11573_T_1/1	8.51	2	2	2	--	37	47	8	--
	GI_L_191_T_3/5	8.04	3	3	4	3	26	37	11	5
	GI_L_399_T_2/6	8.04	4	4	2	--	12	11	4	--
	GI_L_2_T_15/19	6.51	2	2	--	--	12	17	--	--
	GI_L_606_T_2/4	7.98	--	2	--	--	--	10	--	--
Beta-globin	Osmo_L_95_T_3/3	8.71	3	3	3	3	14	15	13	22
Myosin, light chain 12	***GI_L_978_T_1/3***	***4.86***	***3***	***3***	***4***	***3***	***9***	***14***	***29***	***17***
Ezrin	GI_L_1092_T_10/11	8.90	3	2	3	4	21	15	7	17
	Muscle_L_935_T_1/1	10.74	--	--	2	2	--	--	7	9
Erythrocyte band 7 integral membrane protein	GI_L_1045_T_1/2	9.80	3	3	3	3	27	29	10	13
Eosinophil peroxidase	Muscle_L_301_T_24/27	6.99	11	7	3	--	32	18	9	--
Villin-1	***GI_L_741_T_1/3***	***6.79***	***10***	***6***	***4***	***3***	***27***	***12***	***15***	***8***
Protein disulfide-isomerase	GI_L_443_T_5/8	4.55	--	--	6	8	--	--	20	22
Zonadhesin/IgGFc-binding protein	GI_L_7875_T_1/2	5.67	3	3	2	--	14	24	11	--
Prothymosin alpha-B-like	Muscle_L_111_T_10/13	9.11	6	6	2	--	13	14	4	--
Keratin, type I cytoskeletal 18-like	Muscle_L_619_T_2/9	6.85	--	--	8	5	--	--	28	14
	GI_L_307_T_3/12	4.40	--	--	3	--	--	--	5	--
Aspartyl aminopeptidase	GI_L_2939_T_1/1	9.04	2	3	--	3	13	19	--	12
	GI_L_1686_T_5/6	7.21	--	2	--	--	--	2	--	--
Tetratricopeptide repeat protein 38	GI_L_1914_T_2/2	7.27	4	6	--	--	12	22	--	--
Probable aminopeptidase NPEPL1	GI_L_1709_T_1/1	7.04	3	3	5	2	5	10	9	3
Complement component C3	GI_L_1685_T_1/7	6.92	5	5	2	--	14	11	4	--
Selenium-binding protein 1	Neural_L_6082_T_1/1	7.28	4	4	--	2	12	13	--	5
Myosin light polypeptide 6-like	***GI_L_837_T_2/5***	***5.58***	***--***	***--***	***5***	***5***	***--***	***--***	***18***	***14***
Protein SET	Neural_L_6819_T_1/1	4.29	--	--	5	3	--	--	17	22
Alpha-actinin	Muscle_L_15654_T_1/1	3.85	2	--	--	--	6	--	--	--
	Muscle_L_828_T_1/3	***5.04***	***4***	***3***	***2***	***2***	***12***	***6***	***6***	***2***
Angiotensin converting enzyme	GI_L_3242_T_1/1	6.09	2	2	2	--	8	9	3	--
	GI_L_10315_T_1/1	5.77	2	2	--	--	5	7	--	--
	GI_L_323_T_9/13	6.82	2	3	3	2	7	8	7	3
xaa-Pro dipeptidase	Muscle_L_169_T_1/1	6.72	5	4	--	--	10	7	--	--
Aminopeptidase N	GI_L_1506_T_1/2	5.50	8	5	--	--	19	14	--	--
	GI_L_227_T_7/11	6.23	2	2	--	--	7	3	--	--
	GI_L_2417_T_2/6	6.51	2	--	--	--	5	--	--	--
Na/H exchange regulatory cofactor NHE-RF1	GI_L_1017_T_1/1	11.26	--	--	2	--	--	--	9	--

			Unique peptides				Total spectral counts			
			Insoluble		Soluble		Insoluble		Soluble	
Probable protein identification	Accession number	pI	1	2	1	2	1	2	1	2
Pentraxin/Serum amyloid P-component	***GI_L_3606_T_1/1***	***9.70***	***2***	***2***	***--***	***--***	***16***	***13***	***--***	***--***
Apolipoprotein A1-like	Neural_L_10655_T_1/2	7.05	--	--	--	2	--	--	--	6
	Neural_L_623_T_6/7	8.36	--	--	2	3	--	--	11	14
Fish-egg lectin	Muscle_L_514_T_17/21	9.51	2	3	2	--	12	12	2	--
	Muscle_L_514_T_16/21	10.02	2	--	--	--	7	--	--	--
Annexin A5	GI_L_1339_T_4/8	8.82	2	3	5	3	7	4	17	3
Harmonin	GI_L_4494_T_1/1	5.78	--	--	3	3	--	--	12	24
Thioredoxin	GI_L_701_T_1/1	8.51	--	--	7	3	--	--	21	14
Heat shock protein 90-beta	Osmo_L_332_T_21/22	6.66	--	2	--	3	--	6	--	4
Tubulin alpha	Muscle_L_1451_T_2/7	6.65	3	4	--	--	12	17	--	--
Putative aminopeptidase	GI_L_1742_T_1/1	7.15	3	2	--	--	7	9	--	--
Melanotransferrin	GI_L_1587_T_14/14	9.31	2	--	2	--	14	--	12	--
Unknown protein	GI_L_2434_T_11/14	4.84	--	--	3	3	--	--	11	16
NADPH-cytochrome p450 reductase	Muscle_L_342_T_1/1	7.92	--	--	3	2	--	--	14	5
Hemoglobin embryonic subunit alpha-like	GI_L_14273_T_1/1	8.25	--	--	2	2	--	--	4	6
Arginine-tRNA ligase, cytoplasmic	GI_L_587_T_4/5	8.71	3	3	--	--	6	16	--	--
Mucin-2	GI_L_1807_T_18/18	4.68	--	--	5	4	--	--	15	9
Lactate dehydrogenase B	Muscle_L_1741_T_2/3	7.06	3	3	--	--	12	14	--	--
Dipeptidyl peptidase 4	GI_L_484_T_1/1	6.19	2	2	--	--	19	8	--	--
Protein-arginine deiminase	GI_L_952_T_2/4	8.37	2	2	--	2	10	11	--	3
Nattectin	GI_L_147_T_1/5	7.83	2	--	--	--	5	--	--	--
	GI_L_147_T_4/5	7.21	--	2	2	--	--	8	3	--
	Muscle_L_3830_T_4/8	9.40	2	2	--	--	3	9	--	--
Complement C1q tumor necrosis factor-related protein 3	GI_L_13975_T_1/2	10.17	--	--	2	2	--	--	3	10
Cytochrome b5-like	GI_L_2701_T_1/1	7.91	--	--	--	2	--	--	--	3
14-3-3 protein epsilon	Osmo_L_2081_T_1/1	4.38	2	2	3	2	4	7	7	4
Protein disulfide-isomerase A3	GI_L_542_T_3/6	8.67	2	3	2	2	9	8	3	3
Major vault protein	GI_L_751_T_8/11	7.84	4	3	--	--	9	9	--	--
Aldehyde dehydrogenase family 9 member A1	Muscle_L_749_T_1/1	6.99	2	2	--	--	8	10	--	--
Lamin-A	Neural_L_3329_T_1/1	10.03	2	--	2	3	4	--	3	11
Complement C1q tumor necrosis factor	Muscle_L_4117_T_11/12	9.40	2	2	--	--	9	9	--	--
Meprin A subunit beta	***GI_L_2177_T_4/8***	***5.96***	***3***	***2***	***--***	***--***	***18***	***8***	***--***	***--***
Alpha-2-macroglobulin-like protein 1	Neural_L_8297_T_1/1	6.15	2	2	2	2	3	9	6	5
Meprin A subunit alpha	GI_L_1444_T_1/2	5.39	2	2	--	--	8	9	--	--
Puromycin-sensitive aminopeptidase	Neural_L_576_T_1/1	4.79	2	6	--	--	4	13	--	--
Carbonic anhydrase 7	GI_L_12605_T_1/1	9.34	3	2	--	--	7	10	--	--
Heat shock cognate 70kDa protein	Neural_L_309_T_8/9	10.51	4	3	2	--	12	4	3	--
Na/K ATPase subunit beta	GI_L_150_T_10/11	8.30	--	2	--	--	--	3	--	--
Complement component C9	***GI_L_201_T_1/1***	***6.92***	***5***	***4***	***--***	***--***	***7***	***11***	***--***	***--***
Epidermal growth factor receptor kinase substrate 8	GI_L_6924_T_1/1	5.70	--	2	--	--	--	4	--	--
Intestinal-type alkaline phosphotase 1	GI_L_1563_T_7/10	8.29	3	3	--	--	9	5	--	--
Na-K-Cl cotransporter (NKCC2)	GI_L_277_T_26/27	8.49	2	2	--	--	5	5	--	--
Cytosolic nonspecific dipeptidase	GI_L_3393_T_3/4	5.00	--	--	3	2	--	--	5	6
Tropomyosin beta chain	GI_L_2423_T_5/6	5.81	--	--	2	--	--	--	20	--
Tubulin beta	Muscle_L_2134_T_1/2	6.49	4	3	--	--	8	5	--	--
Annexin A1	Neural_L_42_T_3/5	7.10	2	--	2	--	6	--	7	--
Trehalase	Muscle_L_8862_T_1/1	4.69	2	2	2	--	7	3	5	--
Dipeptidase 1	GI_L_3282_T_1/1	8.02	2	--	2	--	4	--	6	--
14-3-3 protein zeta	Muscle_L_47_T_1/2	7.46	--	--	4	2	--	--	6	2
ATP synthase subunit alpha, mitochondrial	Muscle_L_2996_T_1/6	8.61	4	--	2	--	8	--	2	--
14-3-3 protein gamma	Muscle_L_4303_T_1/1	6.52	--	--	2	--	--	--	7	--
Transketolase-like protein 2	Muscle_L_1428_T_19/20	7.91	3	2	--	--	6	3	--	--
Leukotriene A-4 hydrolase	Muscle_L_167_T_1/1	5.64	2	--	--	--	3	--	--	--

			Unique peptides				Total spectral counts			
			Insoluble		Soluble		Insoluble		Soluble	
Probable protein identification	Accession number	pI	1	2	1	2	1	2	1	2
Adrenodoxin, mitochondria	Muscle_L_856_T_1/2	8.63	--	--	2	2	--	--	7	11
Calpain small subunit 1	***Muscle_L_2264_T_1/10***	***7.32***	***--***	***2***	***2***	***2***	***--***	***4***	***3***	***3***
Acidic leucine-rich nuclear phosphoprotein 32 family member B	Muscle_L_5322_T_1/1	3.91	--	--	--	2	--	--	--	6
Prohibitin	GI_L_3375_T_1/1	8.66	2	2	--	--	4	7	--	--
Carboxypeptidase B	GI_L_368_T_2/2	8.28	--	--	2	--	--	--	5	--
Glyceraldehyde-3-phosphate dehydrogenase	Muscle_L_228_T_5/6	10.11	--	--	2	--	--	--	6	--
Annexin A2-B	GI_L_557_T_13/14	8.18	--	--	--	3	--	--	--	9
Guanine nucleotide-binding protein G(q) subunit alpha	Neural_L_10206_T_1/1	5.62	2	2	--	--	6	6	--	--
N-acetylated-alpha-linked acidic dipeptidase	GI_L_2491_T_1/1	8.88	2	--	--	--	4	--	--	--
	GI_L_2491_T_1/1	8.88	2	2	--	--	8	2	--	--
Perforin-1	GI_L_10625_T_1/1	7.16	2	2	--	--	3	8	--	--
Catenin alpha-1	Muscle_L_840_T_1/1	7.99	--	--	--	2	--	--	--	2
Sodium/potassium-transporting ATPase alpha-1	Muscle_L_3299_T_1/1	5.41	4	2	--	--	8	2	--	--
Glutamyl aminopeptidase	GI_L_6564_T_1/1	7.49	--	--	2	--	--	--	6	--
Coagulin factor II/Thrombin	GI_L_563_T_1/2	7.88	--	2	--	--	--	5	--	--
Myosin 11	Muscle_L_573_T_1/1	6.01	--	2	--	2	--	9	--	2
Neutral ceramidase	GI_L_46_T_12/15	7.06	2	--	--	--	4	--	--	--
Unconventional myosin-XV	GI_L_2348_T_1/1	6.52	2	2	--	--	6	6	--	--
Proto-oncogene tyrosine-protein kinase Yrk	Muscle_L_3191_T_1/4	8.82	2	2	--	--	8	5	--	--
Complement Component 1 Q subcomponenet-binding protein, mitochondrial	GI_L_4775_T_1/2	7.83	--	--	--	2	--	--	--	4
Collagen type I alpha 3 chain	Neural_L_2456_T_1/1	8.45	3	--	--	--	9	--	--	--
Leukocyte elastase inhibitor	Muscle_L_3748_T_1/4	7.99	--	--	2	--	--	--	7	--
	Muscle_L_3234_T_3/4	8.75	--	--	4	--	--	--	7	--
	Muscle_L_1016_T_3/5	7.80	--	--	2	2	--	--	3	3
Retinal dehydrogenase 2	Muscle_L_1756_T_1/1	7.48	2	2	--	--	6	5	--	--
Copine 3	***Muscle_L_9022_T_1/1***	***7.45***	***2***	***2***	***--***	***--***	***6***	***6***	***--***	***--***
Fatty acid-binding protein	GI_L_17123_T_1/1	7.62	--	--	2	2	--	--	4	8
Trypsinogen	GI_L_1303_T_8/8	6.75	--	--	2	--	--	--	6	--
Heterogeneous nuclear ribonucleoprotein Q	Muscle_L_346_T_1/1	5.73	--	2	--	--	--	3	--	--
Bile salt-activated lipase	GI_L_1147_T_2/10	7.95	2	2	--	--	4	3	--	--
14-3-3 protein beta/alpha-1	Neural_L_383_T_10/12	6.51	--	--	2	3	--	--	3	5
Glycogen phosphorylase, brain form	Muscle_L_5009_T_1/1	5.67	2	2	--	--	2	6	--	--
ATP synthase subunit beta, mitochondrial	GI_L_1246_T_3/4	7.72	5	--	--	--	5	--	--	--
Complement factor B	Muscle_L_1172_T_1/1	6.30	--	3	--	--	--	5	--	--
Transaldolase	Muscle_L_6930_T_1/1	7.60	--	--	2	--	--	--	3	--
Cadherin 17	***GI_L_2646_T_9/15***	***4.63***	***2***	***--***	***--***	***--***	***3***	***--***	***--***	***--***
Junction plakoglobin	GI_L_1494_T_1/1	9.16	2	2	--	--	3	3	--	--
Coatomer subunit gamma-2	GI_L_3438_T_3/4	5.33	--	3	--	--	--	6	--	--
Alpha-2-HS-glycoprotein	GI_L_79_T_11/18	5.21	--	--	--	2	--	--	--	5
Spectrin beta chain, non-erythrocytic 1	***Osmo_L_101_T_19/21***	***8.01***	***2***	***--***	***2***	***--***	***2***	***--***	***2***	***--***
Actin, alpha cardiac muscle 1-like	Osmo_L_17_T_5/6	9.11	--	2	--	--	--	9	--	--
Elongation factor 2-like	Osmo_L_455_T_5/11	8.00	2	2	--	--	4	4	--	--
Alanine-glyoxylate aminotransferase 2, mitochondrial	Muscle_L_3795_T_1/2	8.85	2	--	--	--	6	--	--	--
Prosaposin	GI_L_1293_T_10/15	7.50	--	--	2	--	--	--	5	--
Creatine kinase U-type, mitochondrial	GI_L_5513_T_3/4	9.39	--	2	--	--	--	4	--	--
Protein NLRC3	Muscle_L_78_T_65/68	7.41	2	--	--	--	4	--	--	--
Phosphoglycerate mutase 1	GI_L_251_T_4/5	9.77	--	2	--	--	--	4	--	--
Calreticulin	Osmo_L_4302_T_1/1	4.35	--	--	2	--	--	--	3	--
Plastin-2	***Muscle_L_2144_T_1/1***	***5.42***	***--***	***3***	***--***	***--***	***--***	***6***	***--***	***--***

			Unique peptides				Total spectral counts			
			Insoluble		Soluble		Insoluble		Soluble	
**Probable protein identification**	**Accession number**	**pI**	**1**	**2**	**1**	**2**	**1**	**2**	**1**	**2**
4-aminobutyrate aminotransferase, mitochondrial	Muscle_L_3168_T_1/3	8.40	--	2	--	--	--	2	--	--
Annexin A14	Neural_L_2415_T_1/1	5.37	--	2	--	--	--	4	--	--
Actin-related protein 3	Muscle_L_5149_T_1/3	6.59	--	2	--	--	--	4	--	--
Glutaredoxin 3	GI_L_2521_T_4/6	7.86	--	2	--	--	--	5	--	--
Maltase-glucoamylase, intestinal	GI_L_3118_T_1/1	6.17	2	--	--	--	3	--	--	--
Collagen alpha-2(I) chain	Neural_L_2064_T_1/1	9.27	3	--	--	--	4	--	--	--
T-complex protein 1 subunit gamma	GI_L_1570_T_5/6	8.67	2	--	--	--	5	--	--	--
Aspartate aminotransferase, mitochondrial	Muscle_L_3356_T_13/14	8.41	2	--	--	--	4	--	--	--
Aspartate-tRNA ligase, cytoplasmic	GI_L_662_T_6/6	8.52	--	3	--	--	--	3	--	--
Staphylococcal nuclease domain-containing protein 1	GI_L_893_T_12/12	7.44	--	2	--	--	--	4	--	--
ATP-dependent RNA helicase	Muscle_L_5236_T_2/4	6.85	--	3	--	--	--	5	--	--
Syntaxin-binding protein 2	Muscle_L_4363_T_1/1	7.11	2	--	--	--	4	--	--	--
Carcinoembryonic antigen-related cell adhesion molecule 1-like	GI_L_73_T_1/1	5.45	--	--	--	2	--	--	--	2
Proactivator polypeptide	GI_L_1273_T_1/2	6.66	--	--	2	--	--	--	2	--
Serine/threonine-protein phosphatase PP1-gamma catalytic subunit A	Muscle_L_2360_T_1/1	8.68	--	3	--	--	--	3	--	--
Tyrosine-protein phosphatase non-receptor type 6	Muscle_L_1776_T_1/2	7.38	2	--	--	--	2	--	--	--
Type-2 ice-structuring protein	Muscle_L_2873_T_8/9	7.41	--	2	--	--	--	3	--	--

Supplementary Table S3: Proteins identified by mass spectrometry in the intestinal fluid as well as the isolated intestinal precipitate matrix purified using the stringent method. Numbers in the table headers denote biological replicates. Proteins in gray are contaminants identified from the cRAP database. Proteins in bold and italics were observed in the matrix samples and contain known calcium binding domains. Accession numbers refer to *de novo* assembled transcripts and have been truncated for clarity.

			Unique peptides					Total spectral counts				
			Intestinal fluid		$CaCO_3$ matrix			Intestinal fluid		$CaCO_3$ matrix		
**Probable protein identification**	**Accession number**	**pI**	**1**	**2**	**1**	**2**	**3**	**1**	**2**	**1**	**2**	**3**
Trypsin (Porcine)	sp|TRYP_PIG|	7.22	7	6	7	8	7	40	39	184	181	194
Keratin, type I cytoskeletal 10 (Human)	sp|K1C10_HUMAN|	4.87	10	5	22	22	17	24	9	96	103	70
Keratin, type II cytoskeletal 1 (Human)	sp|K2C1_HUMAN|	8.34	11	7	22	22	18	33	11	116	124	78
Keratin, type 1 cytoskeletal 9 (Human)	sp|K1C9_HUMAN|	4.95	9	0	13	13	11	14	0	69	66	42
Transferrin	Muscle_L_303_T_1/2	8.06	34	28	--	3	--	124	75	--	9	--
Keratin, type I cytoskeletal 13-like	Muscle_L_968_T_2/2	4.83	--	--	3	--	2	--	--	11	--	4
	Muscle_L_3_T_31/33	5.73	--	--	2	--	--	--	--	7	--	--
	Osmo_L_782_T_2/4	8.50	21	16	3	7	4	88	49	4	32	16
Beta actin	Neural_L_221_T_11/12	8.37	18	21	2	2	2	66	84	6	6	7
	Muscle_L_533_T_1/2	8.17	5	5	--	--	--	67	67	--	--	--
	Muscle_L_24_T_1/8	6.64	2	2	--	--	--	63	66	--	--	--
Apolipoprotein A1-like	Neural_L_623_T_6/7	8.36	15	16	--	--	2	40	38	--	--	3
	Neural_L_10655_T_1/2	7.05	16	13	2	2	2	100	64	5	10	11
Arginine--tRNA ligase, cytoplasmic-like	GI_L_587_T_4/5	8.71	25	33	--	--	--	71	99	--	--	--
Complement component c3	GI_L_1685_T_1/7	6.92	34	32	--	2	2	95	75	--	5	8
	GI_L_1201_T_17/19	7.51	6	2	--	--	--	14	5	--	--	--
	GI_L_5734_T_1/1	8.99	2	--	--	--	--	7	--	--	--	--
	GI_L_1201_T_10/19	8.89	2	3	--	--	--	6	7	--	--	--
Keratin, type II cytoskeletal 8-like	Muscle_L_158_T_1/2	9.27	16	19	4	6	6	34	38	20	37	41
	Osmo_L_130_T_10/14	8.97	16	15	2	4	4	40	34	11	36	35
Alpha-2-macroglobulin-like	GI_L_1225_T_3/8	6.04	31	25	--	--	--	93	68	--	--	--
Keratin, type II cytoskeletal 2 epidermal (Human)	sp|K22E_HUMAN|	8.17	3	2	10	16	13	8	8	41	64	66
NADPH-cytochrome P450 reductase	Muscle_L_342_T_1/1	7.92	--	--	3	4	6	--	--	22	28	89
Protein disulfide-isomerase	GI_L_443_T_5/8	4.55	6	15	3	6	5	15	30	11	22	33
	GI_L_1437_T_1/2	6.45	--	4	--	--	--	--	8	--	--	--
Arginase-1	Muscle_L_144_T_12/14	9.07	12	12	--	--	--	58	54	--	--	--
Thioredoxin	GI_L_701_T_1/1	8.51	8	9	4	4	5	22	28	18	21	29
Protein-arginine deiminase	GI_L_952_T_2/4	8.37	8	11	--	--	--	38	65	--	--	--
Zonadhesin/IgGFc-binding protein	GI_L_7875_T_1/2	5.67	17	12	--	--	3	65	30	--	--	16
Eosinophil peroxidase-like	Muscle_L_301_T_24/27	6.99	34	4	--	--	--	112	6	--	--	--
Cytosolic nonspecific dipeptidase	GI_L_3393_T_3/4	5.00	11	16	3	4	5	24	45	6	7	15
Mucin-2	GI_L_1807_T_18/18	4.68	16	--	3	3	6	46	--	12	11	34
Protein-arginine deiminase type-2	Muscle_L_114_T_35/40	5.22	3	--	--	--	--	37	--	--	--	--
	Muscle_L_114_T_36/40	8.21	22	12	--	--	--	75	27	--	--	--
Creatine kinase B-type	Neural_L_3639_T_7/10	7.57	14	12	--	--	--	47	41	--	--	--
Heat shock cognate 70kDa protein	Osmo_L_15_T_22/26	5.36	12	15	3	2	3	24	32	5	7	9
	Muscle_L_9116_T_1/1	9.14	4	3	--	--	--	22	26	--	--	--
xaa-Pro dipeptidase	Muscle_L_169_T_1/1	6.72	16	15	--	--	--	46	43	--	--	--
Alpha-actinin-4	Muscle_L_828_T_1/3	5.04	19	22	--	--	--	44	46	--	--	--
Calmodulin	***Neural_L_113_T_4/5***	***8.85***	***4***	***4***	***3***	***4***	***3***	***9***	***9***	***5***	***16***	***11***
Immunoglobulin heavy chain	GI_L_4539_T_2/3	4.80	13	12	--	--	--	49	39	--	--	--
Retinal dehydrogenase 2 isoform 1	Muscle_L_1756_T_1/1	7.48	16	14	--	--	--	47	36	--	--	--
Glucose-6-phosphate isomerase	Neural_L_3750_T_1/1	6.95	11	11	--	--	3	29	31	--	--	11
Trypsin	Neural_L_21432_T_1/1	4.38	3	--	2	2	2	5	--	12	22	22
Serum amyloid p-component	GI_L_3606_T_1/1	9.70	3	3	--	--	--	41	40	--	--	--
Angiotensin converting enzyme	GI_L_3242_T_1/1	6.09	23	9	--	--	--	57	18	--	--	--
	GI_L_10315_T_1/1	5.77	7	4	--	--	--	17	10	--	--	--
	GI_L_1318_T_2/3	8.05	2	--	--	--	--	4	--	--	--	--
Lymphocyte cytosolic protein 1 (l-plastin)	Muscle_L_2144_T_1/1	5.42	17	18	--	--	--	36	39	--	--	--
Major vault protein	GI_L_751_T_8/11	7.84	13	17	--	--	--	31	43	--	--	--
Glyceraldehyde-3-phosphate dehydrogenase	Muscle_L_2335_T_3/5	8.10	6	10	--	--	--	23	41	--	--	--
	Muscle_L_228_T_3/6	8.56	6	6	--	--	--	15	17	--	--	--
	GI_L_539_T_3/4	8.46	3	--	--	--	--	9	--	--	--	--
Heat shock protein 90-alpha	Neural_L_955_T_1/2	5.15	3	8	3	3	4	6	18	8	7	12
Enolase 3-2	Muscle_L_6817_T_1/2	9.23	11	11	--	--	--	31	28	--	--	--
	Osmo_L_88_T_6/8	11.42	--	2	--	--	--	--	4	--	--	--
Alpha-1-antitrypsin homolog	Neural_L_2088_T_1/2	7.90	12	6	--	--	--	57	14	--	--	--
Transketolase-like protein 2	Muscle_L_1428_T_19/20	7.91	10	12	--	--	--	29	43	--	--	--
Triosephosphate isomerase	GI_L_1275_T_12/14	10.21	11	12	--	--	--	32	36	--	--	--
14 kda apolipoprotein	Neural_L_5102_T_1/2	6.65	5	4	--	--	--	26	26	--	--	--
Selenium-binding protein 1	Muscle_L_14835_T_1/1	7.50	14	11	--	--	--	37	22	--	--	--
Tetratricopeptide repeat protein 38	GI_L_1914_T_2/2	7.27	14	15	--	--	--	35	35	--	--	--

Probable protein identification	Accession number	pI	Unique peptides: Intestinal fluid 1	Unique peptides: Intestinal fluid 2	Unique peptides: $CaCO_3$ matrix 1	Unique peptides: $CaCO_3$ matrix 2	Unique peptides: $CaCO_3$ matrix 3	Total spectral counts: Intestinal fluid 1	Total spectral counts: Intestinal fluid 2	Total spectral counts: $CaCO_3$ matrix 1	Total spectral counts: $CaCO_3$ matrix 2	Total spectral counts: $CaCO_3$ matrix 3
Glutathione S-transferase	Muscle_L_1836_T_5/6	9.49	7	11	--	--	--	25	29	--	--	--
	GI_L_1104_T_11/12	8.14	4	5	--	--	--	7	8	--	--	--
	Osmo_L_4363_T_1/1	6.25	3	3	--	--	--	13	6	--	--	--
	Neural_L_2854_T_9/12	9.55	2	--	--	--	--	24	--	--	--	--
	GI_L_1104_T_11/12	8.14	2	3	--	--	--	4	7	--	--	--
Alpha-type globin	Osmo_L_343_T_1/3	8.11	4	4	--	--	--	35	23	--	--	--
	Muscle_L_4376_T_1/8	10.22	6	5	--	--	--	22	13	--	--	--
Aldehyde dehydrogenase 9 member a1	Muscle_L_749_T_1/1	6.99	12	14	--	--	--	21	36	--	--	--
Aminopeptidase -like	GI_L_1742_T_1/1	7.15	8	20	--	--	--	13	54	--	--	--
Fructose-bisphosphate aldolase b	Muscle_L_986_T_4/7	7.71	7	11	--	--	--	17	26	--	--	--
Glutathione S-transferase Mu 3-like	Muscle_L_7793_T_3/4	8.08	5	7	2	2	2	11	14	5	10	11
Scinderin like protein a	Muscle_L_4724_T_16/19	9.05	11	13	--	--	--	24	34	--	--	--
	Muscle_L_4724_T_16/19	9.05	2	2	--	--	--	5	5	--	--	--
Tyrosine 3-monooxygenase tryptophan 5-monooxygenase activation epsilon polypeptide	Osmo_L_2081_T_1/1	4.38	8	11	--	--	--	15	20	--	--	--
Curculin domain-containing protein (mannose-binding) lectin	GI_L_1556_T_10/15	6.93	8	11	--	--	--	21	30	--	--	--
Catalase	Osmo_L_2074_T_2/3	8.69	10	13	--	--	--	23	25	--	--	--
Fibrinogen gamma chain	Neural_L_11213_T_1/2	6.03	4	3	--	--	--	19	15	--	--	--
	GI_L_1673_T_10/13	6.68	13	9	--	--	--	35	24	--	--	--
Fibrinogen alpha chain	GI_L_592_T_8/15	9.31	3	4	--	--	--	15	10	--	--	--
	GI_L_4373_T_1/2	5.49	11	8	--	--	--	26	19	--	--	--
Fatty acid binding protein 1	GI_L_4195_T_2/2	4.97	3	4	--	--	--	15	35	--	--	--
Leukotriene a4 hydrolase	Muscle_L_167_T_1/1	5.64	13	12	--	--	--	35	23	--	--	--
Pyruvate kinase	Neural_L_118_T_13/19	8.12	13	12	--	--	--	28	29	--	--	--
Angiotensin converting enzyme 2	GI_L_323_T_9/13	6.82	7	9	--	--	--	16	19	--	--	--
14-3-3 protein beta alpha-2	Muscle_L_543_T_9/15	5.70	8	8	--	--	--	21	27	--	--	--
	Muscle_L_5907_T_1/1	8.23	--	2	--	--	--	--	8	--	--	--
Glutathione s-transferase theta-1	Muscle_L_1454_T_10/11	9.21	8	9	--	--	--	22	25	--	--	--
Malate cytoplasmic	Muscle_L_16697_T_1/1	9.14	10	9	--	--	--	28	24	--	--	--
Peroxiredoxin 1	GI_L_3253_T_3/4	7.39	6	7	--	--	--	15	16	--	--	--
	Muscle_L_859_T_1/2	8.41	4	4	--	--	--	12	10	--	--	--
ATP synthase subunit beta, mitochondrial	GI_L_1246_T_3/4	7.72	--	12	3	2	2	--	25	4	5	7
Glutamyl aminopeptidase	GI_L_6564_T_1/1	7.49	11	6	--	--	--	34	17	--	--	--
	GI_L_1842_T_1/1	8.37	8	6	--	--	--	13	9	--	--	--
	GI_L_1842_T_1/1	8.37	2	2	--	--	--	7	5	--	--	--
Peroxiredoxin 6	Neural_L_880_T_1/1	6.98	6	7	--	--	--	18	22	--	--	--
Hemoglobin beta chain	Osmo_L_95_T_3/3	8.71	2	2	--	--	--	41	21	--	--	--
	Osmo_L_1419_T_1/2	8.87	4	4	--	--	--	25	23	--	--	--
Aminopeptidase-like 1	GI_L_1709_T_1/1	7.04	9	10	--	--	--	22	26	--	--	--
Beta 2c	Muscle_L_2134_T_1/2	6.49	4	4	--	--	--	8	22	--	--	--
Trypsinogen	GI_L_3370_T_1/2	4.88	--	--	2	2	3	--	--	9	8	14
Sialate o-acetylesterase-like	GI_L_2847_T_1/1	5.60	8	4	--	--	--	31	12	--	--	--
Ubiquitin and ribosomal protein 27a precursor	GI_L_13_T_3/9	10.81	2	3	--	--	--	3	7	--	--	--
Uncharacterized protein	GI_L_555_T_1/3	8.97	8	6	--	--	--	31	11	--	--	--
Fructose-bisphosphate aldolase c	Neural_L_1178_T_7/8	9.93	9	8	--	--	--	24	22	--	--	--
Alpha skeletal muscle	Osmo_L_266_T_11/17	6.50	2	--	--	--	--	49	--	--	--	--
Protein disulfide isomerase family member 3	Muscle_L_3345_T_1/2	8.09	8	13	--	--	--	12	26	--	--	--
Phospholipase A2	GI_L_659_T_1/5	6.32	--	--	2	2	2	--	--	7	7	10
Keratin, type I cytoskeletal 18-like	Muscle_L_619_T_2/9	6.85	5	7	--	2	3	12	13	--	2	5
Aminopeptidase puromycin sensitive	Neural_L_576_T_1/1	4.79	10	9	--	--	--	23	19	--	--	--
Inter-alpha-trypsin inhibitor heavy chain h3-like	GI_L_2308_T_4/10	7.61	13	5	--	--	--	32	12	--	--	--
	GI_L_3433_T_7/8	5.56	3	2	--	--	--	12	10	--	--	--
	GI_L_397_T_2/11	4.05	2	2	--	--	--	6	4	--	--	--
	GI_L_1716_T_1/1	4.87	3	--	--	--	--	5	--	--	--	--
C-1-tetrahydrofolate cytoplasmic	GI_L_884_T_6/6	10.58	7	11	--	--	--	14	24	--	--	--
	GI_L_884_T_6/6	10.58	3	6	--	--	--	4	15	--	--	--
Lactate dehydrogenase B	Neural_L_264_T_1/2	9.28	7	6	--	--	2	14	14	--	--	5
Protein SET	Neural_L_6819_T_1/1	4.29	2	3	--	--	3	4	6	--	--	16

			Unique peptides					Total spectral counts				
			Intestinal fluid		$CaCO_3$ matrix			Intestinal fluid		$CaCO_3$ matrix		
**Probable protein identification**	**Accession number**	**pI**	**1**	**2**	**1**	**2**	**3**	**1**	**2**	**1**	**2**	**3**
Gdp dissociation inhibitor 1	Muscle_L_6631_T_1/1	5.45	8	9	--	--	--	19	17	--	--	--
Peptidyl arginine type ii	Muscle_L_10300_T_1/1	6.62	6	8	--	--	--	12	24	--	--	--
Beta-hexosaminidase subunit beta	GI_L_2936_T_1/2	9.51	14	4	--	--	--	28	8	--	--	--
Phosphoglycerate kinase 1	Neural_L_219_T_1/2	7.66	9	7	--	--	--	23	20	--	--	--
Adenylyl cyclase-associated protein 1	GI_L_1938_T_10/12	7.79	8	8	--	2	2	18	15	--	3	4
Complement component c9	GI_L_201_T_1/1	6.92	11	5	--	--	--	32	7	--	--	--
Alpha-2-macroglobulin-like protein 1-like	Neural_L_3714_T_1/2	5.00	5	5	--	--	--	81	70	--	--	--
	Neural_L_8297_T_1/1	6.15	2	2	--	--	--	11	3	--	--	--
Chymotrypsin B-like	GI_L_1336_T_3/11	8.19	6	6	--	2	3	13	11	--	2	4
Melanotransferrin precursor	GI_L_1587_T_14/14	10.10	8	4	--	--	--	20	10	--	--	--
	GI_L_1587_T_14/14	10.10	2	--	--	--	--	10	--	--	--	--
GDP-mannose 4,6 dehydratase	GI_L_1430_T_1/1	7.18	3	6	--	--	2	9	13	--	--	9
Meprin a subunit alpha	GI_L_1444_T_1/2	5.39	11	3	--	--	--	24	9	--	--	--
	GI_L_983_T_2/5	6.59	9	3	--	--	--	19	10	--	--	--
Hemoglobin embryonic subunit alpha-	GI_L_14273_T_1/1	8.25	5	5	--	--	--	16	12	--	--	--
Tributyltin binding protein type 2	Neural_L_1346_T_6/9	6.54	8	5	--	--	--	21	12	--	--	--
3 (2) -bisphosphate nucleotidase 1	GI_L_1410_T_1/1	6.51	6	13	--	--	--	12	26	--	--	--
Cytosolic sulfotransferase 3	Muscle_L_929_T_1/1	8.84	9	5	--	--	--	18	10	--	--	--
Annexin a1	Neural_L_42_T_3/5	7.10	9	9	--	--	--	21	19	--	--	--
Elastase-1	GI_L_1537_T_1/2	4.29	2	--	2	2	2	4	--	5	5	5
	GI_L_3096_T_12/15	8.16	3	5	--	--	--	8	8	--	--	--
Type-iv antifreeze protein	Neural_L_14023_T_1/1	4.69	6	6	--	--	--	19	18	--	--	--
Myosin-9	Muscle_L_3751_T_1/1	6.64	6	7	--	--	--	10	15	--	--	--
Adrenodoxin, mitochondrial	Muscle_L_856_T_1/2	8.63	--	--	--	--	2	--	--	--	--	7
Meprin a subunit beta	GI_L_1634_T_14/18	7.02	4	--	--	--	--	8	--	--	--	--
	GI_L_2177_T_4/8	5.96	12	5	--	--	--	22	9	--	--	--
Enolase alpha non-neuron	Muscle_L_1959_T_1/2	10.05	7	5	--	--	--	22	17	--	--	--
Cytosolic 10-formyltetrahydrofolate dehydrogenase	GI_L_2248_T_5/11	5.65	6	11	--	--	--	11	23	--	--	--
	GI_L_332_T_9/13	7.63	3	6	--	--	--	5	12	--	--	--
	Osmo_L_3270_T_1/1	8.79	--	2	--	--	--	--	13	--	--	--
Alpha-2-macroglobulin precursor	Neural_L_14155_T_1/1	8.27	5	4	--	--	--	35	24	--	--	--
Fatty acyl- hydrolase medium chain-like	Muscle_L_2831_T_3/4	7.75	2	12	--	--	--	3	26	--	--	--
	Muscle_L_630_T_1/2	4.87	2	4	--	--	--	4	10	--	--	--
Tributyltin binding protein type 1	Neural_L_11577_T_1/1	8.66	9	5	--	--	--	22	7	--	--	--
	GI_L_184_T_15/17	4.71	2	--	--	--	--	15	--	--	--	--
14-3-3 protein gamma-2	Muscle_L_4303_T_1/1	6.52	--	6	--	2	2	--	19	--	7	8
Adenosylhomocysteinase	Muscle_L_1264_T_14/15	9.78	6	8	--	--	--	11	17	--	--	--
Gelsolin (finnish type)	Neural_L_2163_T_1/1	5.00	4	7	--	--	--	9	17	--	--	--
Atp synthase subunit alpha, mitochondrial-like	Osmo_L_963_T_1/2	9.61	--	8	--	--	--	--	23	--	--	--
Alpha-2-hs-glycoprotein precursor	Neural_L_12238_T_1/1	6.03	6	6	--	--	--	11	14	--	--	--
Fetuin-b-like	GI_L_159_T_3/6	7.61	6	5	--	--	--	16	14	--	--	--
	GI_L_159_T_6/6	8.54	4	3	--	--	--	21	10	--	--	--
Peptidyl-prolyl cis-trans isomerase-like	GI_L_241_T_8/10	9.90	5	5	--	--	--	13	17	--	--	--
Complement factor partial	Neural_L_9784_T_1/1	8.48	7	7	--	--	--	36	29	--	--	--
	Neural_L_9784_T_1/1	8.48	4	--	--	--	--	15	--	--	--	--
Hemopexin-like	GI_L_1975_T_10/13	8.08	4	5	--	--	--	21	10	--	--	--
	GI_L_3317_T_1/2	7.60	3	2	--	--	--	7	4	--	--	--
Immunoglobulin mu heavy chain	Muscle_L_69_T_228/229	9.50	9	4	--	--	--	31	18	--	--	--
Serpin peptidase clade a (alpha-1 antitrypsin) member 7	GI_L_1072_T_19/26	7.33	6	5	--	--	--	17	9	--	--	--
Tribytitin bining protein type 1-like	GI_L_5001_T_2/6	6.02	4	4	--	--	--	27	12	--	--	--
	GI_L_1151_T_1/1	6.61	3	--	--	--	--	16	--	--	--	--
Cystatin-b	Muscle_L_3433_T_2/9	10.13	4	4	--	--	--	11	17	--	--	--
3 -phosphoadenosine 5 -phosphosulfate synthase 2	Muscle_L_4271_T_1/1	8.50	10	4	--	--	--	24	8	--	--	--
Transgelin	Muscle_L_931_T_6/11	10.41	5	7	--	--	--	10	17	--	--	--
Nucleoside diphosphate kinase	Neural_L_1547_T_1/6	8.50	4	5	--	--	--	10	12	--	--	--

Probable protein identification	Accession number	pI	Unique peptides					Total spectral counts				
			Intestinal fluid		$CaCO_3$ matrix			Intestinal fluid		$CaCO_3$ matrix		
			1	2	1	2	3	1	2	1	2	3
Acidic mammalian chitinase-like	GI_L_2796_T_1/2	8.65	2	2	--	--	--	14	8	--	--	--
	GI_L_2_T_15/19	6.51	5	4	--	--	--	11	9	--	--	--
	GI_L_2277_T_3/7	10.17	3	2	--	--	--	8	5	--	--	--
	GI_L_191_T_3/5	8.04	4	--	--	--	--	7	--	--	--	--
	GI_L_2_T_15/19	6.51	2	--	--	--	--	7	--	--	--	--
	GI_L_399_T_1/6	8.05	2	--	--	--	--	3	--	--	--	--
Uncharacterized protein	GI_L_1107_T_5/8	8.41	6	5	--	--	--	15	16	--	--	--
Prosaposin	GI_L_1293_T_10/15	7.50	4	3	--	2	--	6	4	--	3	--
	GI_L_1273_T_1/2	6.66	3	2	--	--	--	10	6	--	--	--
Plastin-1	GI_L_449_T_1/1	6.81	5	8	--	--	--	14	23	--	--	--
Trypsin-2-like	GI_L_6439_T_2/2	5.53	2	--	--	--	2	3	--	--	--	3
C1 inhibitor	GI_L_1683_T_3/4	6.54	8	3	--	--	--	20	6	--	--	--
C-factor-like	GI_L_4981_T_1/1	8.01	6	6	--	--	--	13	14	--	--	--
Argininosuccinate lyase	Muscle_L_3325_T_3/4	8.19	5	9	--	--	--	10	17	--	--	--
Alcohol dehydrogenase class-3	Muscle_L_4413_T_1/2	8.90	4	7	--	--	--	8	16	--	--	--
Abhydrolase domain-containing protein 14b	Muscle_L_5199_T_1/1	8.80	6	5	--	--	--	14	14	--	--	--
Cadherin-related family member 2	GI_L_3470_T_13/13	4.13	6	4	--	--	--	20	10	--	--	--
	GI_L_12089_T_1/1	4.22	3	3	--	--	--	7	6	--	--	--
Proteasome activator complex subunit 1	Neural_L_12570_T_1/1	6.52	3	8	--	--	--	6	19	--	--	--
Hemoglobin subunit beta-2	Osmo_L_7524_T_1/1	9.68	7	6	--	--	--	21	11	--	--	--
Histone	GI_L_1008_T_2/5	11.06	2	--	--	--	--	7	--	--	--	--
	Muscle_L_15998_T_1/1	10.29	2	2	--	--	--	3	6	--	--	--
Leucine-rich alpha-2-glycoprotein-like	GI_L_10357_T_1/2	6.46	5	--	--	--	--	18	--	--	--	--
Elongation factor 1-alpha	Neural_L_311_T_1/4	10.12	2	--	2	--	--	4	--	3	--	--
	Muscle_L_626_T_5/7	9.52	--	2	--	--	--	--	4	--	--	--
Type-2 ice-structuring	GI_L_110_T_1/8	8.68	5	2	--	--	--	15	3	--	--	--
	Muscle_L_16411_T_1/1	7.53	2	--	--	--	--	3	--	--	--	--
	Muscle_L_2873_T_8/9	7.26	2	--	--	--	--	3	--	--	--	--
Pancreatic elastase	GI_L_108_T_1/9	8.95	6	4	--	--	--	15	9	--	--	--
Profilin-2	Muscle_L_145_T_34/35	8.54	4	7	--	--	--	11	19	--	--	--
	GI_L_1343_T_3/7	8.41	--	3	--	--	--	--	4	--	--	--
Complement c4-like	Muscle_L_238_T_4/8	7.28	12	--	--	--	--	20	--	--	--	--
Ependymin-like	Muscle_L_4059_T_2/6	10.04	2	2	--	--	2	7	4	--	--	9
Unknown Protein	Muscle_L_6293_T_1/4	6.84	2	--	--	2	--	9	--	--	8	--
Gastrotropin-like	GI_L_2656_T_1/3	9.54	4	4	--	--	--	10	12	--	--	--
Aminopeptidase N	GI_L_96_T_5/6	9.42	5	3	--	--	--	18	10	--	--	--
	GI_L_296_T_23/24	6.58	2	2	--	--	--	14	8	--	--	--
	GI_L_1506_T_1/2	5.50	8	--	--	--	--	16	--	--	--	--
	Muscle_L_23623_T_1/1	4.38	4	2	--	--	--	9	3	--	--	--
	Muscle_L_21936_T_1/1	4.75	4	3	--	--	--	17	10	--	--	--
	GI_L_13364_T_1/1	6.07	2	2	--	--	--	5	6	--	--	--
Fibrinogen beta chain precursor	Neural_L_15207_T_1/1	8.75	6	4	--	--	--	16	8	--	--	--
Lumican precursor	Neural_L_3160_T_1/2	7.73	7	4	--	--	--	13	12	--	--	--
Elongation factor 2	Osmo_L_455_T_9/11	9.34	3	8	--	--	--	7	16	--	--	--
	Osmo_L_1055_T_3/4	8.03	2	2	--	--	--	3	6	--	--	--
Annexin a5	GI_L_1339_T_4/8	8.82	12	--	--	--	--	24	--	--	--	--
Aldehyde dehydrogenase 2 family	Osmo_L_584_T_1/1	5.40	--	10	--	--	--	--	25	--	--	--
Transaldolase	Muscle_L_6930_T_1/1	7.60	5	7	--	--	--	11	15	--	--	--
Warm temperature acclimation protein 65 kda-2	GI_L_1640_T_4/7	6.84	4	3	--	--	--	10	6	--	--	--
Angiotensinogen	GI_L_6386_T_1/1	5.42	7	4	--	--	--	21	10	--	--	--
Fatty acid-binding protein, intestinal-like	GI_L_6731_T_1/1	6.55	4	4	--	--	--	12	17	--	--	--
Actin-related protein 3	Muscle_L_5149_T_1/3	6.59	4	5	--	--	--	9	12	--	--	--
Heavy chain non-muscle	Muscle_L_98_T_1/2	5.17	4	9	--	--	--	7	17	--	--	--
Ubiquitin-like modifier-activating enzyme 1	Muscle_L_1838_T_1/1	6.03	2	8	--	--	--	8	15	--	--	--
Dipeptidase 1-like	GI_L_3282_T_1/1	8.02	8	2	--	--	--	18	3	--	--	--
14-3-3 protein gamma	Muscle_L_47_T_1/2	7.46	4	4	--	--	--	11	10	--	--	--
Beta-globin, partial	GI_L_7027_T_1/1	8.49	5	5	--	--	--	16	13	--	--	--
Chymotrypsin-like protease CTRL-1	GI_L_9227_T_2/2	9.20	6	4	--	--	--	16	7	--	--	--

Probable protein identification	Accession number	pI	Unique peptides: Intestinal fluid 1	Unique peptides: Intestinal fluid 2	Unique peptides: $CaCO_3$ matrix 1	Unique peptides: $CaCO_3$ matrix 2	Unique peptides: $CaCO_3$ matrix 3	Total spectral counts: Intestinal fluid 1	Total spectral counts: Intestinal fluid 2	Total spectral counts: $CaCO_3$ matrix 1	Total spectral counts: $CaCO_3$ matrix 2	Total spectral counts: $CaCO_3$ matrix 3
Olfactomedin-4	GI_L_1046_T_4/5	4.73	5	--	--	--	--	9	--	--	--	--
	GI_L_2299_T_1/1	6.25	4	2	--	--	--	21	5	--	--	--
	GI_L_3403_T_4/10	6.37	5	--	--	--	--	12	--	--	--	--
	GI_L_24_T_4/6	7.77	4	3	--	--	--	12	5	--	--	--
Dipeptidyl peptidase 4-like	GI_L_484_T_1/1	6.19	6	5	--	--	--	14	11	--	--	--
	GI_L_3008_T_1/1	4.56	2	--	--	--	--	8	--	--	--	--
Type i cytoskeletal 18	GI_L_307_T_11/12	4.51	6	6	--	--	--	12	15	--	--	--
Maltase-glucoamylase, intestinal-like	GI_L_3118_T_1/1	6.17	4	4	--	--	--	14	7	--	--	--
Carbonyl reductase	GI_L_345_T_9/12	9.34	7	4	--	--	--	13	7	--	--	--
Proteasome subunit alpha type-7-like	GI_L_3940_T_1/1	8.52	5	7	--	--	--	10	14	--	--	--
Nucleolin-like isoform 2	Muscle_L_1714_T_1/1	4.80	3	6	--	--	--	7	18	--	--	--
Sulfotransferase 6b1	Muscle_L_3419_T_1/1	7.29	5	4	--	--	--	10	10	--	--	--
Actin binding 1c	Muscle_L_3719_T_1/1	7.05	5	4	--	--	--	13	8	--	--	--
	Muscle_L_3188_T_1/1	6.45	2	3	--	--	--	4	9	--	--	--
Argininosuccinate synthase	Muscle_L_970_T_1/5	8.27	3	3	--	--	--	10	10	--	--	--
Fructose- -bisphosphatase 1	Neural_L_565_T_1/2	9.00	5	5	--	--	--	10	12	--	--	--
Kininogen 1	Neural_L_18613_T_1/1	4.74	3	3	--	--	--	9	8	--	--	--
Tubulin alpha-1a chain	Muscle_L_1451_T_2/7	6.65	5	3	--	--	--	11	8	--	--	--
60s ribosomal protein l7	Osmo_L_218_T_1/2	11.26	3	6	--	--	--	6	15	--	--	--
Glucosamine-6-phosphate isomerase 1	GI_L_4444_T_1/1	6.88	6	4	--	--	--	16	9	--	--	--
Catenin alpha-1	Muscle_L_840_T_1/1	7.99	3	--	--	--	2	3	--	--	--	3
Unknown Protein	Muscle_L_12311_T_1/1	7.74	--	--	2	2	2	--	--	5	8	9
Alpha-2-HS-glycoprotein	GI_L_79_T_17/18	7.44	4	2	--	--	2	8	5	--	--	7
Ependymin-2-like	GI_L_1362_T_12/18	7.05	3	--	--	--	2	5	--	--	--	5
Calreticulin precursor	Osmo_L_4302_T_1/1	4.35	2	7	--	--	--	4	13	--	--	--
Calreticulin precursor	GI_L_1300_T_2/2	4.24	--	2	--	--	--	--	17	--	--	--
Methylthioadenosine phosphorylase	Muscle_L_1512_T_1/1	8.16	3	5	--	--	--	9	13	--	--	--
Wd repeat-containing protein 1	Muscle_L_2272_T_1/2	8.25	--	7	--	--	--	--	16	--	--	--
Ceruloplasmin	GI_L_1565_T_1/1	6.69	6	3	--	--	--	14	6	--	--	--
60S acidic ribosomal protein P2	GI_L_3276_T_1/1	4.48	3	3	--	--	--	7	9	--	--	--
Xaa-pro aminopeptidase 2	GI_L_10900_T_1/1	5.89	7	3	--	--	--	17	5	--	--	--
	GI_L_5437_T_1/1	5.88	2	--	--	--	--	3	--	--	--	--
Pyridoxal kinase	Muscle_L_754_T_1/1	5.53	5	4	--	--	--	12	12	--	--	--
Tropomyosin alpha-4 chain-like	GI_L_360_T_5/8	6.55	3	3	--	--	2	9	10	--	--	3
Amiloride-sensitive amine oxidase	GI_L_841_T_11/12	8.57	4	4	--	--	--	6	7	--	--	--
Cystathionine gamma-lyase	Muscle_L_3689_T_2/5	8.42	3	5	--	--	--	7	10	--	--	--
Sh3 domain-binding glutamic acid-rich-like protein	Osmo_L_6404_T_1/1	9.05	4	5	--	--	--	9	11	--	--	--
Proteasome activator complex subunit 2	Muscle_L_4237_T_1/1	5.57	3	5	--	--	--	4	11	--	--	--
Erythrocyte band 7 integral membrane protein	Muscle_L_1404_T_1/1	6.09	7	--	--	--	--	14	--	--	--	--
Cytochrome c	Muscle_L_5093_T_1/5	10.09	6	6	--	--	--	11	11	--	--	--
Xaa-pro aminopeptidase 1	GI_L_2266_T_6/8	6.78	3	5	--	--	--	6	10	--	--	--
Uncharacterized protein	GI_L_4274_T_1/1	7.22	--	6	--	--	--	--	12	--	--	--
Coagulin factor ii	GI_L_563_T_2/2	10.01	6	2	--	--	--	14	3	--	--	--
Trypsin inhibitor CITI-1-like	GI_L_8172_T_1/1	6.32	--	--	--	2	2	--	--	--	5	5
Gdp-mannose -dehydratase	Muscle_L_10793_T_1/2	11.32	2	2	--	--	--	5	14	--	--	--
Dipeptidyl peptidase 3	Muscle_L_9013_T_1/1	5.62	2	5	--	--	--	4	13	--	--	--
Tropomyosin alpha-1 chain	GI_L_298_T_6/13	4.59	3	5	--	--	--	4	8	--	--	--
Isocitrate dehydrogenase	Muscle_L_1976_T_1/2	8.14	5	5	--	--	--	8	11	--	--	--
Proteasome subunit alpha type-2	Muscle_L_6280_T_1/1	7.80	4	4	--	--	--	7	10	--	--	--
Cofilin-2	Muscle_L_880_T_3/5	10.18	2	4	--	--	--	4	11	--	--	--
Transitional endoplasmic reticulum atpase	Muscle_L_1461_T_1/2	5.61	2	6	--	--	--	4	13	--	--	--
Plasminogen	GI_L_1605_T_3/5	7.88	7	--	--	--	--	16	--	--	--	--
	GI_L_7024_T_1/1	8.56	3	--	--	--	--	5	--	--	--	--
Beta-galactoside-binding lectin	Muscle_L_203_T_14/21	9.20	5	--	--	--	--	20	--	--	--	--
Granulins precursor	Muscle_L_1584_T_4/5	7.89	7	--	--	--	--	15	--	--	--	--
Nidogen 2 precursor	GI_L_7203_T_3/4	6.37	5	3	--	--	--	10	9	--	--	--
Creatine kinase U-type, mitochondrial-like	GI_L_5513_T_3/4	9.39	2	6	--	--	--	4	12	--	--	--
Neoverrucotoxin subunit alpha-like	Muscle_L_3089_T_1/1	5.69	--	5	--	--	--	--	17	--	--	--

Probable protein identification	Accession number	pI	Unique peptides: Intestinal fluid 1	Unique peptides: Intestinal fluid 2	Unique peptides: $CaCO_3$ matrix 1	Unique peptides: $CaCO_3$ matrix 2	Unique peptides: $CaCO_3$ matrix 3	Total spectral counts: Intestinal fluid 1	Total spectral counts: Intestinal fluid 2	Total spectral counts: $CaCO_3$ matrix 1	Total spectral counts: $CaCO_3$ matrix 2	Total spectral counts: $CaCO_3$ matrix 3
Complement C1q tumor necrosis factor-related protein 3-like	GI_L_13975_T_1/2	10.17	--	--	--	--	2	--	--	--	--	8
Uncharacterized protein	GI_L_649_T_5/5	4.47	3	--	--	--	--	5	--	--	--	--
Glycogen liver form	Muscle_L_2454_T_2/3	7.97	5	2	--	--	--	10	4	--	--	--
Glutathione s-transferase omega 1	Neural_L_18388_T_1/1	10.14	--	2	--	--	--	--	4	--	--	--
	GI_L_7146_T_1/1	6.95	5	3	--	--	--	9	6	--	--	--
Moesin	Muscle_L_3140_T_1/4	9.58	3	4	--	--	--	7	10	--	--	--
Phosphoglycerate mutase 1	GI_L_251_T_4/5	9.77	3	2	--	--	--	7	5	--	--	--
Fibrinogen beta chain	GI_L_357_T_3/5	7.04	3	2	--	--	--	10	6	--	--	--
	GI_L_357_T_4/5	8.61	3	--	--	--	--	10	--	--	--	--
D-amino-acid oxidase	GI_L_1163_T_1/1	8.07	2	3	--	--	--	6	9	--	--	--
Zeta (quinone reductase)	Neural_L_12780_T_1/1	4.93	2	3	--	--	--	8	13	--	--	--
Adipose differentiation-related protein	GI_L_2504_T_1/1	8.29	2	3	--	--	--	3	14	--	--	--
Histone h2b type 1-b-like	Muscle_L_10726_T_1/1	11.17	--	2	--	--	--	--	10	--	--	--
Pdzk1l protein	Muscle_L_5010_T_1/1	6.64	7	--	--	--	--	16	--	--	--	--
Chymotrypsin-like elastase family member 2A	GI_L_331_T_1/1	10.25	2	2	--	--	--	7	4	--	--	--
Neutral ceramidase	GI_L_46_T_10/15	6.79	5	2	--	--	--	10	4	--	--	--
N-myc downstream regulated gene 1	Muscle_L_1563_T_4/5	9.09	3	2	--	--	--	6	12	--	--	--
Acyl-coenzyme A thioesterase 4-like	GI_L_7895_T_1/1	6.46	3	4	--	--	--	7	7	--	--	--
Proteasome subunit alpha type-5	GI_L_5247_T_1/1	5.43	2	5	--	--	--	5	10	--	--	--
Zgc:171352 protein	GI_L_885_T_7/9	6.22	6	2	--	--	--	14	4	--	--	--
Aspartate cytoplasmic	GI_L_1048_T_4/6	9.47	5	2	--	--	--	9	3	--	--	--
Glutathione peroxidase	GI_L_2436_T_1/2	10.07	3	--	--	--	--	5	--	--	--	--
Prothymosin alpha-B-like	GI_L_2434_T_11/14	4.84	--	--	2	2	2	--	--	4	5	8
Myeloperoxidase	Muscle_L_11067_T_1/1	6.60	3	--	--	--	--	7	--	--	--	--
Legumain	Muscle_L_741_T_1/1	7.29	3	2	--	--	--	6	4	--	--	--
Carboxypeptidase b	Neural_L_11538_T_1/1	9.14	3	3	--	--	--	7	5	--	--	--
	Neural_L_11538_T_1/1	9.14	2	--	--	--	--	3	--	--	--	--
60s ribosomal protein l10a	Muscle_L_247_T_4/4	10.51	3	4	--	--	--	5	6	--	--	--
Aspartyl aminopeptidase	GI_L_2939_T_1/1	9.04	3	5	--	--	--	6	8	--	--	--
Cathepsin h	GI_L_1751_T_1/2	7.85	6	--	--	--	--	16	--	--	--	--
Receptor tyrosine-kinase erB-2	GI_L_3099_T_1/4	6.77	5	3	--	--	--	12	6	--	--	--
40s ribosomal protein s18	Neural_L_1193_T_1/1	11.41	4	5	--	--	--	6	11	--	--	--
Ester hydrolase c11orf54 homolog	Muscle_L_13453_T_1/1	6.69	3	3	--	--	--	8	7	--	--	--
Heterogeneous nuclear ribonucleoprotein q isoform 1	Muscle_L_346_T_1/1	5.73	2	3	--	--	--	8	9	--	--	--
Enterokinase light chain	GI_L_2454_T_1/1	8.23	3	2	--	--	--	9	6	--	--	--
Gamma-glutamyltranspeptidase 1-like	Muscle_L_490_T_5/6	8.60	6	--	--	--	--	13	--	--	--	--
Villin 1	GI_L_741_T_1/3	6.79	3	--	--	--	--	6	--	--	--	--
Uncharacterized protein	GI_L_1828_T_7/9	8.04	2	2	--	--	--	8	3	--	--	--
Calpastatin long	Muscle_L_424_T_9/18	4.61	4	2	--	--	--	7	2	--	--	--
Complement factor h	Neural_L_15777_T_1/2	7.11	4	--	--	--	--	5	--	--	--	--
Complement factor H-like	GI_L_4159_T_3/3	7.71	5	--	--	--	--	8	--	--	--	--
Lactate dehydrogenase-a	Muscle_L_327_T_7/8	7.44	2	3	--	--	--	7	10	--	--	--
Citrate synthase	Muscle_L_5843_T_6/7	9.99	3	3	--	--	--	6	6	--	--	--
Ribosomal protein l6	Neural_L_2083_T_1/3	11.17	3	5	--	--	--	5	12	--	--	--
Tbt-binding partial	GI_L_828_T_1/6	8.15	4	2	--	--	--	6	7	--	--	--
Fumarylacetoacetate hydrolase domain-containing protein 2a	Muscle_L_16203_T_1/1	6.61	4	2	--	--	--	13	4	--	--	--
Inosine-uridine preferring nucleoside hydrolase-like	Muscle_L_19197_T_1/1	6.11	4	2	--	--	--	12	3	--	--	--
Cadherin-17	GI_L_2646_T_9/15	4.63	4	--	--	--	--	9	--	--	--	--
Carboxypeptidase a1 precursor	Muscle_L_2725_T_1/2	6.86	3	2	--	--	--	8	3	--	--	--
Thioredoxin reductase 3	Muscle_L_1835_T_1/1	7.07	--	5	--	--	--	--	10	--	--	--
Annexin a2-a	Muscle_L_4371_T_1/1	5.76	4	4	--	--	--	8	8	--	--	--
	Muscle_L_15108_T_1/1	7.88	5	--	--	--	--	8	--	--	--	--
Malate mitochondrial-like	Osmo_L_356_T_1/2	8.50	2	6	--	--	--	5	9	--	--	--
Glycogen brain form	Muscle_L_5009_T_1/1	5.67	2	3	--	--	--	8	8	--	--	--
Endoplasmin precursor	Muscle_L_8194_T_1/1	4.50	--	4	--	--	--	--	16	--	--	--
Complement factor b-like	Muscle_L_1172_T_1/1	6.30	7	--	--	--	--	13	--	--	--	--
Activated RNA polymerase II transcriptional coactivator p15	Muscle_L_7571_T_1/1	10.06	--	--	--	--	2	--	--	--	--	8

			Unique peptides					Total spectral counts				
			Intestinal fluid		$CaCO_3$ matrix			Intestinal fluid		$CaCO_3$ matrix		
**Probable protein identification**	**Accession number**	**pI**	**1**	**2**	**1**	**2**	**3**	**1**	**2**	**1**	**2**	**3**
Alcohol dehydrogenase 1	GI_L_3029_T_6/8	8.49	4	3	--	--	--	6	5	--	--	--
Fatty acid-binding protein, liver	GI_L_4194_T_2/2	4.07	3	3	--	--	--	6	13	--	--	--
Alpha-aspartyl dipeptidase	GI_L_1203_T_2/4	9.78	4	2	--	--	--	8	6	--	--	--
F-box only protein 50	GI_L_4993_T_1/1	5.10	3	2	--	--	--	7	3	--	--	--
Scavenger receptor cysteine-rich type 1 protein M130-like	GI_L_2882_T_1/2	7.46	3	2	--	--	--	8	6	--	--	--
Ethylmalonic encephalopathy 1	Osmo_L_1353_T_1/4	8.60	2	5	--	--	--	4	10	--	--	--
Carbonic anhydrase	Muscle_L_229_T_20/23	8.84	3	--	--	--	--	9	--	--	--	--
Serine threonine-protein phosphatase 2a 65 kda regulatory subunit a alpha isoform	Neural_L_3236_T_1/2	7.03	--	4	--	--	--	--	11	--	--	--
Clathrin heavy chain 1	Muscle_L_3268_T_1/1	5.33	--	5	--	--	--	--	12	--	--	--
	Muscle_L_3235_T_1/1	8.02	--	5	--	--	--	--	7	--	--	--
NADP-dependent malic enzyme-like	GI_L_5297_T_7/13	8.68	--	7	--	--	--	--	14	--	--	--
Tetraspanin-8-like	GI_L_5758_T_1/2	10.48	--	--	--	--	2	--	--	--	--	6
4-aminobutyrate aminotransferase	Muscle_L_3168_T_1/3	8.40	--	4	--	--	--	--	7	--	--	--
Perforin-1	GI_L_2076_T_5/8	9.65	3	--	--	--	--	7	--	--	--	--
	GI_L_3785_T_1/10	4.80	2	--	--	--	--	4	--	--	--	--
Proteasome subunit beta type-3	Muscle_L_18182_T_1/1	6.11	3	2	--	--	--	6	3	--	--	--
Bifunctional protein ncoat-like	GI_L_742_T_1/2	5.89	4	3	--	--	--	9	4	--	--	--
Myosin light polypeptidase 6	GI_L_837_T_3/5	4.10	3	3	--	--	--	5	5	--	--	--
Aspartate mitochondrial precursor	Muscle_L_3356_T_11/14	7.87	4	4	--	--	--	8	7	--	--	--
Phosphatidylethanolamine-binding protein variant 1	Neural_L_9165_T_1/1	8.97	4	3	--	--	--	7	6	--	--	--
Non-histone chromosomal HMG-14A-	Neural_L_4081_T_1/2	9.42	2	3	--	--	--	5	8	--	--	--
Prothrombin isoform 1	GI_L_162_T_10/16	7.01	4	2	--	--	--	8	4	--	--	--
Ribokinase	Muscle_L_14026_T_1/1	7.98	2	2	--	--	--	5	8	--	--	--
Cell division cycle 42 (gtp binding 25kda)	Neural_L_4959_T_9/10	9.77	2	2	--	--	--	8	3	--	--	--
Calcium calmodulin-dependent protein kinase ii delta partial	GI_L_2160_T_1/1	7.69	3	2	--	--	--	7	5	--	--	--
Sulfotransferase 1c1-like	GI_L_1495_T_2/6	7.49	3	--	--	--	--	7	--	--	--	--
60S ribosomal L23a	GI_L_194_T_1/3	9.84	--	2	--	--	--	--	6	--	--	--
Retinol-binding protein 2-like protein	GI_L_1192_T_1/1	8.49	--	4	--	--	--	--	9	--	--	--
S100-a1	Osmo_L_8656_T_1/2	6.04	2	--	--	--	--	4	--	--	--	--
Alanine aminotransferase 1	GI_L_4030_T_8/11	8.81	3	3	--	--	--	5	8	--	--	--
60s ribosomal protein l13	Muscle_L_132_T_2/9	12.03	3	3	--	--	--	5	5	--	--	--
Cathepsin b precursor	GI_L_1432_T_5/7	7.70	3	3	--	--	--	5	7	--	--	--
	GI_L_274_T_3/4	5.28	2	--	--	--	--	4	--	--	--	--
Rho gdp dissociation inhibitor alpha	Osmo_L_1018_T_1/1	6.26	2	3	--	--	--	4	7	--	--	--
Cold-inducible rna-binding protein	GI_L_2222_T_5/6	10.24	2	2	--	--	--	4	6	--	--	--
Arsenite methyltransferase	GI_L_6853_T_1/1	5.32	--	4	--	--	--	--	10	--	--	--
N -dimethylarginine dimethylaminohydrolase 1	Neural_L_3385_T_1/1	7.48	--	2	--	--	--	--	7	--	--	--
Ribosomal protein s3	Osmo_L_2052_T_4/6	9.68	--	4	--	--	--	--	8	--	--	--
Leukocyte cell-derived chemotaxin 2 precursor	GI_L_429_T_1/6	10.26	5	--	--	--	--	12	--	--	--	--
	Neural_L_6871_T_1/1	8.83	2	--	--	--	--	4	--	--	--	--
60 kda heat shock mitochondrial-like	Muscle_L_2283_T_1/1	6.82	--	6	--	--	--	--	11	--	--	--
Adp-ribosylation factor 1	Neural_L_2513_T_1/2	7.48	--	2	--	--	--	--	4	--	--	--
Iq motif containing gtpase activating protein 1	Muscle_L_3152_T_1/4	6.84	3	2	--	--	--	4	3	--	--	--
Serine hydroxymethyltransferase, cytosolic	GI_L_4880_T_1/1	6.26	3	2	--	--	--	5	5	--	--	--
	GI_L_4328_T_1/1	9.00	3	2	--	--	--	4	4	--	--	--
EH domain-containing protein 1-like	GI_L_6832_T_1/1	7.19	2	4	--	--	--	4	5	--	--	--
Ribosomal protein s6	Muscle_L_84_T_8/11	11.96	3	2	--	--	--	4	3	--	--	--
Elongation factor 1-gamma	Muscle_L_2369_T_1/2	7.80	2	4	--	--	--	4	8	--	--	--
Filamin-a isoform 2	Muscle_L_627_T_1/3	8.76	4	2	--	--	--	8	2	--	--	--
Heat shock protein 67b2	GI_L_655_T_3/4	8.88	2	2	--	--	--	6	7	--	--	--
6- liver type	Muscle_L_2661_T_2/2	8.90	3	--	--	--	--	6	--	--	--	--
Alkaline phosphatase	GI_L_956_T_10/13	8.67	4	--	--	--	--	10	--	--	--	--
Complement component c6	Muscle_L_9232_T_1/1	7.92	2	--	--	--	--	7	--	--	--	--
	Muscle_L_9232_T_1/1	7.92	2	--	--	--	--	4	--	--	--	--
40S ribosomal protein S19	GI_L_8049_T_1/1	10.97	--	5	--	--	--	--	10	--	--	--

Probable protein identification	Accession number	pI	Unique peptides					Total spectral counts				
			Intestinal fluid		$CaCO_3$ matrix			Intestinal fluid		$CaCO_3$ matrix		
			1	2	1	2	3	1	2	1	2	3
Mitochondrial hydroxyacyl-coenzyme a dehydrogenase	Muscle_L_13044_T_1/4	8.63	--	3	--	--	--	--	10	--	--	--
Galectin-2	Muscle_L_13869_T_1/1	9.09	--	3	--	--	--	--	8	--	--	--
Attractin	Neural_L_30_T_3/4	7.19	6	--	--	--	--	12	--	--	--	--
Lactase-phlorizin hydrolase-like	GI_L_8055_T_1/1	4.49	--	2	--	--	--	--	2	--	--	--
60s acidic ribosomal protein p0	Muscle_L_692_T_1/2	8.12	--	4	--	--	--	--	8	--	--	--
High choriolytic enzyme 1 precursor	GI_L_2247_T_10/12	7.80	4	--	--	--	--	8	--	--	--	--
Sex hormone-bing globulin	GI_L_9986_T_1/1	4.72	3	2	--	--	--	8	3	--	--	--
40s ribosomal protein sa	Muscle_L_2908_T_1/7	7.06	2	4	--	--	--	4	8	--	--	--
Uncharacterized protein	GI_L_10171_T_1/1	6.72	3	--	--	--	--	6	--	--	--	--
Ezrin	GI_L_1092_T_10/11	8.90	2	3	--	--	--	4	6	--	--	--
Alpha 1b	Muscle_L_4504_T_13/14	9.96	--	4	--	--	--	--	9	--	--	--
Ribosomal protein s9	GI_L_716_T_10/11	11.09	--	2	--	--	--	--	5	--	--	--
Carbonic anhydrase 4-like	Muscle_L_1921_T_6/10	7.96	3	--	--	--	--	10	--	--	--	--
Alkaline phosphatase-like	GI_L_3567_T_7/8	7.86	3	--	--	--	--	12	--	--	--	--
Gtp-binding nuclear protein ran	GI_L_787_T_7/8	10.13	--	4	--	--	--	--	8	--	--	--
Cub and zona pellucida-like domain-containing protein 1-like	Muscle_L_111_T_10/13	9.11	5	--	--	--	--	11	--	--	--	--
	Muscle_L_2460_T_1/6	8.45	2	--	--	--	--	4	--	--	--	--
Isochorismatase domain-containing 2, mitochondrial	GI_L_3066_T_1/1	7.96	2	2	--	--	--	5	4	--	--	--
Uncharacterized protein	GI_L_10652_T_1/1	5.77	2	--	--	--	--	3	--	--	--	--
40s ribosomal protein s5	Muscle_L_5024_T_1/1	10.47	--	3	--	--	--	--	6	--	--	--
Aldose reductase	Osmo_L_6653_T_1/1	6.42	3	4	--	--	--	4	7	--	--	--
6-phosphogluconate decarboxylating	Muscle_L_693_T_6/8	8.47	2	2	--	--	--	5	4	--	--	--
Intestinal-type alkaline phosphatase 1-like	GI_L_1563_T_7/10	8.29	4	--	--	--	--	7	--	--	--	--
Apoptosis-associated speck-like protein containing a card	Muscle_L_4274_T_1/2	9.03	--	3	--	--	--	--	6	--	--	--
	Muscle_L_11384_T_1/1	8.64	--	2	--	--	--	--	3	--	--	--
Thymosin beta-b	Osmo_L_8309_T_1/2	8.09	--	2	--	--	--	--	5	--	--	--
Biliverdin reductase a	Muscle_L_2731_T_1/1	7.44	--	3	--	--	--	--	7	--	--	--
Inter-alpha-trypsin inhibitor heavy chain h2	GI_L_1132_T_1/1	8.97	3	--	--	--	--	8	--	--	--	--
78 kda glucose-regulated	Muscle_L_6165_T_1/2	5.56	--	3	--	--	--	--	5	--	--	--
Cadherin-related family member 5	GI_L_1885_T_3/11	5.37	4	--	--	--	--	9	--	--	--	--
Mannose-binding lectin-associated serine protease-3b	GI_L_5198_T_1/1	8.13	4	--	--	--	--	6	--	--	--	--
Zonadhesin precursor	GI_L_706_T_2/5	6.77	4	--	--	--	--	9	--	--	--	--
60 kda heat shock mitochondrial precursor	Muscle_L_21256_T_1/1	4.48	--	4	--	--	--	--	7	--	--	--
40s ribosomal protein s2	Osmo_L_1319_T_1/2	10.61	--	4	--	--	--	--	9	--	--	--
Catechol-o-methyltransferase domain-containing protein 1	GI_L_55_T_16/17	8.68	3	2	--	--	--	6	4	--	--	--
Hephaestin-like protein 1	Muscle_L_5975_T_1/1	5.81	2	--	--	--	--	4	--	--	--	--
Unknown Protein	GI_L_1412_T_1/1	11.06	2	2	--	--	--	5	4	--	--	--
Light chain 12	GI_L_978_T_1/3	4.86	2	3	--	--	--	4	5	--	--	--
Alpha-actinin-1 isoform partial	Muscle_L_15654_T_1/1	3.85	2	2	--	--	--	5	4	--	--	--
Carcinoembryonic antigen-related cell adhesion molecule 5-like	Muscle_L_4769_T_2/6	8.63	2	--	--	--	--	4	--	--	--	--
N-acetylated-alpha-linked acidic dipeptidase-like protein	GI_L_2491_T_1/1	8.88	4	--	--	--	--	7	--	--	--	--
	GI_L_2491_T_1/1	8.88	3	--	--	--	--	6	--	--	--	--
Alpha-2-antiplasmin	GI_L_2857_T_1/1	7.16	4	--	--	--	--	6	--	--	--	--
Apolipoprotein e	Neural_L_7257_T_1/2	8.37	2	--	--	--	--	4	--	--	--	--
Actin-related protein 2 3 complex subunit 2	Muscle_L_1008_T_1/2	8.81	2	--	--	--	--	4	--	--	--	--
40s ribosomal protein s25-like	Muscle_L_557_T_1/6	10.54	--	2	--	--	--	--	4	--	--	--
Beta-2-glycoprotein 1-like	GI_L_2384_T_3/6	8.34	3	--	--	--	--	6	--	--	--	--
Arp2 actin-related protein 2 homolog	Muscle_L_1025_T_1/1	6.13	--	3	--	--	--	--	5	--	--	--
Ribosomal protein l7a	Muscle_L_794_T_1/10	11.16	--	5	--	--	--	--	7	--	--	--
Phosphoglucomutase 1	Osmo_L_154_T_4/12	9.29	4	--	--	--	--	8	--	--	--	--
Epidermal growth factor receptor kinase substrate 8-like protein 2	GI_L_5581_T_1/1	6.99	2	--	--	--	--	4	--	--	--	--
	GI_L_1133_T_3/4	9.34	4	--	--	--	--	7	--	--	--	--
T-complex protein 1 subunit gamma	GI_L_1570_T_5/6	8.67	--	5	--	--	--	--	10	--	--	--
Uncharacterized protein	Muscle_L_14589_T_1/1	4.30	4	--	--	--	--	7	--	--	--	--

Probable protein identification	Accession number	pI	Unique peptides					Total spectral counts				
			Intestinal fluid		$CaCO_3$ matrix			Intestinal fluid		$CaCO_3$ matrix		
			1	2	1	2	3	1	2	1	2	3
Heterogeneous nuclear ribonucleoprotein a b	Muscle_L_3896_T_1/1	8.00	--	5	--	--	--	--	10	--	--	--
	Muscle_L_368_T_2/4	8.13	--	4	--	--	--	--	9	--	--	--
Inorganic pyrophosphatase	Neural_L_960_T_1/1	6.26	--	4	--	--	--	--	7	--	--	--
Unknown Protein	GI_L_10107_T_1/1	7.36	--	--	--	--	--	--	--	--	--	--
Protocadherin Fat 4-like	GI_L_6319_T_1/1	4.00	2	--	--	--	--	7	--	--	--	--
Proteasome subunit beta type-4	Neural_L_14116_T_1/1	5.10	3	3	--	--	--	4	5	--	--	--
Ribosomal protein l18	GI_L_1269_T_16/18	10.96	2	2	--	--	--	5	4	--	--	--
S-formylglutathione hydrolase	GI_L_1323_T_2/3	8.57	2	2	--	--	--	3	5	--	--	--
Neuroblast differentiation-associated protein ahnak	Muscle_L_617_T_12/20	10.25	--	2	--	--	--	--	4	--	--	--
Immunoglobulin light chain	GI_L_2004_T_1/1	4.43	4	--	--	--	--	8	--	--	--	--
L-rhamnose-binding lectin csl2-like	Muscle_L_2_T_1/2	8.31	4	--	--	--	--	8	--	--	--	--
Spectrin alpha brain	Neural_L_2043_T_3/3	5.71	2	--	--	--	--	6	--	--	--	--
Glutathione reductase	Muscle_L_12388_T_1/1	8.97	2	--	--	--	--	6	--	--	--	--
	Muscle_L_22005_T_1/1	8.69	--	2	--	--	--	--	3	--	--	--
Stress-induced-phosphoprotein 1	Neural_L_1720_T_1/1	6.60	--	4	--	--	--	--	6	--	--	--
Synaptic vesicle membrane protein vat-1 homolog	GI_L_2513_T_1/3	10.77	--	2	--	--	--	--	5	--	--	--
Cartilage intermediate layer 2-like	GI_L_10519_T_3/3	3.99	3	--	--	--	--	7	--	--	--	--
Unknown Protein	Muscle_L_233_T_1/3	6.75	--	--	--	2	--	--	--	--	5	--
Alpha-2-macroglobulin 1	GI_L_10587_T_1/1	5.38	2	--	--	--	--	18	--	--	--	--
CD59B glycoprotein-like	GI_L_3214_T_4/4	8.28	3	--	--	--	--	7	--	--	--	--
Amine oxidase	GI_L_1435_T_10/14	7.48	--	4	--	--	--	--	7	--	--	--
Ectonucleotide pyrophosphatase phosphodiesterase family member 7-like	GI_L_635_T_9/12	9.08	3	--	--	--	--	6	--	--	--	--
Cathepsin l-like	GI_L_965_T_21/23	7.27	5	--	--	--	--	8	--	--	--	--
Syntenin-1-like	GI_L_3169_T_4/4	10.53	2	--	--	--	--	9	--	--	--	--
T-complex protein 1 subunit alpha-like	Muscle_L_2095_T_1/2	6.88	--	2	--	--	--	--	3	--	--	--
Unknown Protein	GI_L_4281_T_2/3	7.64	2	2	--	--	--	4	4	--	--	--
15-hydroxyprostaglandin dehydrogenase [NAD(+)]-like	GI_L_5722_T_1/2	8.78	2	2	--	--	--	3	5	--	--	--
Phosphoenolpyruvate carboxykinase	Muscle_L_4501_T_1/1	8.98	2	2	--	--	--	3	4	--	--	--
	GI_L_2773_T_1/1	7.78	--	3	--	--	--	--	5	--	--	--
Phosphomannomutase 2	Muscle_L_2419_T_1/1	4.99	2	--	--	--	--	3	--	--	--	--
Kh domain rna signal transduction associated 1	Muscle_L_1762_T_6/7	10.67	2	2	--	--	--	3	4	--	--	--
Proteasome subunit alpha type-4	GI_L_11554_T_1/1	8.63	2	--	--	--	--	4	--	--	--	--
Harmonin	GI_L_4494_T_1/1	5.78	3	--	--	--	--	5	--	--	--	--
Na(+) h(+) exchange regulatory cofactor nhe-rf1-like	GI_L_1017_T_1/1	11.26	2	--	--	--	--	3	--	--	--	--
Pseudechetoxin-like protein precursor	Muscle_L_22428_T_1/1	5.59	3	--	--	--	--	5	--	--	--	--
Migration and invasion enhancer 1	GI_L_3994_T_2/6	8.62	3	--	--	--	--	5	--	--	--	--
Trypsinogen 3	GI_L_3957_T_2/8	7.42	2	--	--	--	--	5	--	--	--	--
Programmed cell death 6 interacting protein	Muscle_L_2063_T_1/1	5.42	--	3	--	--	--	--	6	--	--	--
Udp-n-acetylhexosamine pyrophosphorylase	GI_L_2265_T_1/1	6.45	--	3	--	--	--	--	4	--	--	--
Ribosomal protein l15	Muscle_L_2644_T_1/2	12.16	--	2	--	--	--	--	4	--	--	--
Ribosomal protein s16	Neural_L_2689_T_3/3	10.69	--	3	--	--	--	--	5	--	--	--
Translationally-controlled tumor protein	Neural_L_3145_T_3/9	7.38	--	3	--	--	--	--	6	--	--	--
Cysteine and glycine-rich protein 1	Muscle_L_600_T_7/8	8.95	2	--	--	--	--	4	--	--	--	--
Immunoglobulin m heavy chain secreted partial	GI_L_1175_T_5/6	7.43	2	--	--	--	--	10	--	--	--	--
Ribonuclease UK114	GI_L_11297_T_1/2	8.59	--	4	--	--	--	--	7	--	--	--
Protocadherinn gamma-B2-like	GI_L_3967_T_3/6	4.31	4	--	--	--	--	7	--	--	--	--
Kallikrein-8 precursor	Muscle_L_2124_T_1/1	7.18	3	--	--	--	--	6	--	--	--	--
Cytochrome p450 3a	Muscle_L_2572_T_2/8	7.91	--	3	--	--	--	--	4	--	--	--
Ribosomal protein l9	Muscle_L_798_T_10/15	10.47	--	3	--	--	--	--	6	--	--	--
Zonadhesin precursor	GI_L_706_T_4/5	6.76	3	--	--	--	--	6	--	--	--	--
Oxygen-dependent coproporphyrinogen-III oxidase, mitochondrial	Neural_L_3733_T_1/1	8.30	--	--	--	--	2	--	--	--	--	6
Nascent polypeptide-associated complex subunit alpha	GI_L_485_T_2/7	8.04	--	2	--	--	--	--	5	--	--	--

			Unique peptides					Total spectral counts				
			Intestinal fluid		$CaCO_3$ matrix			Intestinal fluid		$CaCO_3$ matrix		
**Probable protein identification**	**Accession number**	**pI**	**1**	**2**	**1**	**2**	**3**	**1**	**2**	**1**	**2**	**3**
Intestinal-typ fatty acid-binding protein	GI_L_5212_T_2/7	10.97	2	--	--	--	--	7	--	--	--	--
40s ribosomal protein s4	Neural_L_91_T_6/8	8.78	--	2	--	--	--	--	4	--	--	--
Poly	Neural_L_9762_T_1/1	9.05	--	2	--	--	--	--	8	--	--	--
Copine i	Muscle_L_3141_T_1/4	5.26	2	--	--	--	--	7	--	--	--	--
Heat shock protein 90	Muscle_L_17_T_36/39	5.19	--	2	--	--	--	--	16	--	--	--
Proteasome subunit beta type-1	GI_L_8828_T_1/1	6.31	2	2	--	--	--	3	4	--	--	--
Prolyl endopeptidase	Muscle_L_10224_T_1/1	5.66	--	2	--	--	--	--	3	--	--	--
Proteasome subunit beta type-1-a	Muscle_L_6796_T_1/1	5.95	--	2	--	--	--	--	3	--	--	--
Acid sphingomelinase-like phoshphodiesterase 3b	GI_L_13140_T_1/1	9.20	2	--	--	--	--	5	--	--	--	--
Chloride intracellular channel 1	Muscle_L_6047_T_1/1	8.01	3	--	--	--	--	4	--	--	--	--
Rna 3 -terminal phosphate cyclase-like protein	Muscle_L_1401_T_14/15	8.01	2	--	--	--	--	4	--	--	--	--
Unknown Protein	GI_L_11393_T_1/1	9.42	2	--	--	--	--	4	--	--	--	--
Actin-related 2/3 complex subunit 4	GI_L_3733_T_1/2	9.48	--	3	--	--	--	--	5	--	--	--
Glycerol-3-phosphate dehydrogenase 1-like	Muscle_L_5942_T_1/2	8.66	--	2	--	--	--	--	4	--	--	--
Glycerol-3-phosphate dehydrogenase [NAD(+)], cytoplasmic	GI_L_4986_T_1/5	8.43	--	2	--	--	--	--	4	--	--	--
Betaine--homocysteine s-methyltransferase 1	GI_L_389_T_1/1	8.73	2	--	--	--	--	3	--	--	--	--
	Osmo_L_1063_T_3/4	8.06	--	2	--	--	--	--	5	--	--	--
Heterogenous nuclear ribonucleoprotein K	GI_L_5077_T_2/2	8.89	--	3	--	--	--	--	4	--	--	--
Myotrophin	GI_L_721_T_1/1	6.50	--	2	--	--	--	--	3	--	--	--
Hyaluronan-binding protein 2	GI_L_30_T_3/5	5.32	2	--	--	--	--	6	--	--	--	--
	GI_L_2947_T_1/1	5.04	2	--	--	--	--	5	--	--	--	--
Acidic (leucine-rich) nuclear phosphoprotein 32 member b	Muscle_L_457_T_2/4	4.37	--	3	--	--	--	--	3	--	--	--
40S ribosomal protein S13, partial	GI_L_546_T_1/3	11.17	--	2	--	--	--	--	4	--	--	--
Annexin a4	GI_L_1356_T_1/1	6.79	3	--	--	--	--	5	--	--	--	--
Annexin a13	Neural_L_2415_T_1/1	5.37	3	--	--	--	--	7	--	--	--	--
Heterogeneous nuclear ribonucleoprotein a0	Neural_L_2880_T_1/1	8.49	--	2	--	--	--	--	4	--	--	--
Eh-domain containing 2	Neural_L_6142_T_1/1	9.26	3	--	--	--	--	5	--	--	--	--
Uncharacterized protein	GI_L_6148_T_1/2	9.47	--	2	--	--	--	--	7	--	--	--
40S ribosomal S8	GI_L_502_T_1/7	10.62	--	2	--	--	--	--	3	--	--	--
Nucleobindin-2 precursor	***Muscle_L_7507_T_1/1***	***6.06***	***--***	***--***	***--***	***2***	***--***	***--***	***--***	***--***	***4***	***--***
Ras-related protein rab-7a	GI_L_1324_T_1/1	6.49	2	--	--	--	--	4	--	--	--	--
Filamin-A-like	GI_L_5067_T_1/1	6.08	2	--	--	--	--	4	--	--	--	--
Lambda-crystallin homolog	GI_L_3163_T_3/5	6.94	2	--	--	--	--	3	--	--	--	--
Member ras oncogene family	Neural_L_3021_T_1/1	9.57	--	2	--	--	--	--	4	--	--	--
14-3-3 protein beta alpha	Neural_L_3527_T_1/1	6.28	--	2	--	--	--	--	8	--	--	--
Unknown Protein	Muscle_L_2814_T_1/1	10.00	--	2	--	--	--	--	4	--	--	--
Histone h5a	Osmo_L_2028_T_1/2	11.38	--	2	--	--	--	--	4	--	--	--
Ras-related c3 botulinum toxin substrate 2 precursor	Muscle_L_538_T_24/31	10.06	2	--	--	--	--	5	--	--	--	--
Glutamate dehydrogenase	GI_L_1599_T_1/1	8.79	--	4	--	--	--	--	4	--	--	--
Vinculin	Muscle_L_14047_T_1/1	5.80	--	3	--	--	--	--	3	--	--	--
Polymerase i and transcript release factor	Muscle_L_1861_T_6/7	8.09	3	--	--	--	--	5	--	--	--	--
Lamin a c	Neural_L_3329_T_1/1	10.03	--	3	--	--	--	--	5	--	--	--
Nadp-dependent malic mitochondrial-like	Osmo_L_3403_T_1/1	7.21	--	2	--	--	--	--	4	--	--	--
Ribosomal protein l17	Neural_L_878_T_1/7	10.64	--	2	--	--	--	--	3	--	--	--
Glutaredoxin-3	GI_L_2521_T_2/6	10.97	2	--	--	--	--	5	--	--	--	--
N-acetylglucosamine-6-sulfatase precursor	Neural_L_1989_T_4/5	8.51	2	--	--	--	--	3	--	--	--	--
Chaperonin containing subunit 6a (zeta 1)	GI_L_1026_T_9/11	6.82	--	2	--	--	--	--	5	--	--	--
Serine protease inhibitor A3K-like	GI_L_5369_T_3/5	8.07	2	--	--	--	--	6	--	--	--	--
Uncharacterized protein	GI_L_7421_T_1/1	4.90	2	--	--	--	--	4	--	--	--	--
Endoplasmic reticulum aminopeptidase	Muscle_L_16997_T_1/1	6.72	--	2	--	--	--	--	6	--	--	--
Adipocyte plasma membrane-associated protein	Muscle_L_3899_T_1/2	9.27	--	2	--	--	--	--	5	--	--	--

Probable protein identification	Accession number	pI	Unique peptides: Intestinal fluid 1	Unique peptides: Intestinal fluid 2	Unique peptides: $CaCO_3$ matrix 1	Unique peptides: $CaCO_3$ matrix 2	Unique peptides: $CaCO_3$ matrix 3	Total spectral counts: Intestinal fluid 1	Total spectral counts: Intestinal fluid 2	Total spectral counts: $CaCO_3$ matrix 1	Total spectral counts: $CaCO_3$ matrix 2	Total spectral counts: $CaCO_3$ matrix 3
Cytochrome family subfamily polypeptide 1	GI_L_957_T_48/51	8.50	--	2	--	--	--	--	4	--	--	--
40s ribosomal protein s7	Osmo_L_1151_T_1/2	11.09	--	2	--	--	--	--	3	--	--	--
Cathepsin z precursor	Muscle_L_7067_T_2/2	8.22	2	--	--	--	--	3	--	--	--	--
PTB domain-containing engulfment adapter protein 1-like	GI_L_7618_T_1/1	9.68	2	--	--	--	--	4	--	--	--	--
Glia maturation factor gamma	Muscle_L_1945_T_1/2	9.97	2	--	--	--	--	3	--	--	--	--
Septin 2	Muscle_L_1961_T_1/2	7.88	--	2	--	--	--	--	2	--	--	--
Proteasome (macropain) 26s non- 2	Neural_L_6517_T_1/2	7.89	--	2	--	--	--	--	3	--	--	--
Small ubiquitin-related modifier 3	GI_L_8137_T_1/1	7.05	--	2	--	--	--	--	3	--	--	--
78 kda glucose-regulated protein precursor	Osmo_L_4852_T_1/1	5.22	--	2	--	--	--	--	3	--	--	--
Actin-related 2/3 complex subunit 5	GI_L_6142_T_1/1	10.40	--	2	--	--	--	--	3	--	--	--
Rho-related gtp-binding	Osmo_L_3376_T_1/2	9.97	2	--	--	--	--	2	--	--	--	--
Arf-gap with dual ph domain-containing protein 1-like	Muscle_L_9080_T_1/1	9.64	2	--	--	--	--	3	--	--	--	--
60s ribosomal protein l12	Neural_L_2362_T_1/5	10.14	--	2	--	--	--	--	3	--	--	--
Caspase-3 precursor	Muscle_L_3973_T_1/1	7.41	--	2	--	--	--	--	4	--	--	--
Lamin b2	Muscle_L_4359_T_1/1	9.29	--	3	--	--	--	--	5	--	--	--
C4b-binding protein alpha chain precursor	Muscle_L_5325_T_3/4	7.77	3	--	--	--	--	5	--	--	--	--
Sulfide:quinone mitochondrial	Muscle_L_6010_T_1/2	9.40	--	3	--	--	--	--	5	--	--	--
Guanine deaminase	Muscle_L_2181_T_1/1	6.74	--	3	--	--	--	--	5	--	--	--
S-adenosylhomocysteine hydrolase-like 1	Muscle_L_4066_T_1/1	7.35	--	2	--	--	--	--	4	--	--	--
Staphylococcal nuclease domain-containing protein 1	GI_L_893_T_12/12	7.44	--	2	--	--	--	--	3	--	--	--
3-ketoacyl- mitochondrial-like	Neural_L_12784_T_1/1	6.76	--	2	--	--	--	--	5	--	--	--
Tropomyosin alpha-3 chain	GI_L_744_T_1/1	4.85	--	2	--	--	--	--	3	--	--	--
Apoptosis-inducing factor 1, mitochondrial	GI_L_5348_T_1/1	6.24	--	2	--	--	--	--	3	--	--	--
ADP/ATP translocase 2	GI_L_3564_T_1/1	10.33	--	2	--	--	--	--	3	--	--	--
Insulin-like growth factor-binding protein complex acid labile subunit	GI_L_11733_T_1/1	7.91	2	--	--	--	--	4	--	--	--	--
Long-chain-fatty-acid-- ligase 5	GI_L_123_T_35/42	8.97	--	2	--	--	--	--	4	--	--	--
Sepiapterin reductase-like	GI_L_1245_T_1/8	8.20	--	2	--	--	--	--	4	--	--	--
Methylmalonate-semialdehyde dehydrogenase	Muscle_L_1180_T_1/1	7.22	--	2	--	--	--	--	4	--	--	--
Uncharacterized protein	Muscle_L_21946_T_1/1	4.26	2	--	--	--	--	3	--	--	--	--
Ewing sarcoma breakpoint region 1	Muscle_L_8189_T_3/6	9.78	--	2	--	--	--	--	4	--	--	--
Stress-70 mitochondrial	Neural_L_654_T_1/2	9.34	--	2	--	--	--	--	4	--	--	--
Glutaredoxin-1	Neural_L_1190_T_1/1	9.70	2	--	--	--	--	4	--	--	--	--
Peroxiredoxin-4	GI_L_3164_T_1/4	5.93	--	2	--	--	--	--	4	--	--	--
Serine threonine kinase 24	Muscle_L_5058_T_1/2	9.57	--	2	--	--	--	--	2	--	--	--
Nicotinamide phosphoribosyltransferase	GI_L_13657_T_1/1	9.74	--	2	--	--	--	--	2	--	--	--
A-kinase anchor protein 8-like	Muscle_L_7648_T_1/1	10.15	--	2	--	--	--	--	3	--	--	--
Alpha-galactosidase a	Muscle_L_8088_T_1/2	6.93	2	--	--	--	--	3	--	--	--	--
Intestinal mucin-like	GI_L_3381_T_1/1	6.13	2	--	--	--	--	2	--	--	--	--
3-oxoacid transferase 1	Osmo_L_1261_T_1/1	6.52	--	2	--	--	--	--	3	--	--	--
Heat schock cognate 71 kDa protein	GI_L_7714_T_1/1	6.95	--	2	--	--	--	--	24	--	--	--
Cd2-associated protein	Muscle_L_10646_T_1/1	10.29	--	2	--	--	--	--	3	--	--	--
Rna-binding protein 4b	Neural_L_1466_T_1/1	10.09	--	2	--	--	--	--	2	--	--	--
Asparaginyl-trna cytoplasmic-like	Muscle_L_3576_T_1/2	8.05	--	2	--	--	--	--	2	--	--	--
Chloride intracellular channel 5	Muscle_L_5883_T_1/1	9.57	--	2	--	--	--	--	2	--	--	--
Arylsulfatase a	GI_L_231_T_3/4	7.23	2	--	--	--	--	2	--	--	--	--
Myosin ic	Muscle_L_5257_T_1/1	9.77	--	2	--	--	--	--	2	--	--	--

Supplementary Table S4: Composition of the salines used in the micro-modified *in vitro* calcification assay, pH-stat, and electron microscopy (EM) experiments. For the pH-stat and EM experiments the thymol blue was omitted. The calcium buffer, bicarbonate buffer, and TBS (containing matrix proteins if appropriate) were mixed to result in a final ratio of 9:9:2 in the assays.

Salt	Concentration in calcium buffer	Concentration in bicarbonate buffer	Concentration in TBS	Final assay concentration	Standard curve buffer
NaCl	60 mM	60 mM	150 mM	69 mM	69 mM
KCl	5.56 mM	5.56 mM	--	5 mM	5 mM
$MgSO_4$	86.11 mM	86.11 mM	--	77.5 mM	77.5 mM
$MgCl_2$	25 mM	25 mM	--	22.5 mM	22.5 mM
$NaHCO_3$	--	55.56 mM	--	25 mM	25 mM
$CaCl_2$	44.44 mM	--	--	20 mM	20 mM
Tris Base	--	--	10 mM	1 mM	100 mM
Thymol Blue	0.0044%	0.0044%	--	0.004%	0.004%
pH	8.6	8.6	7.5	--	--

Supplementary References

1 Zerbino, D. R. & Birney, E. Velvet: algorithms for de novo short read assembly using de Bruijn graphs. *Genome Res.* (2008).

2 Schulz, M. H., Zerbino, D. R., Vingron, M. & Birney, E. Oases: robust de novo RNA-seq assembly across the dynamic range of expression levels. *Bioinformatics* **28**, 1086-1092 (2012).

3 Conesa, A. *et al.* Blast2GO: a universal tool for annotation, visualization and analysis in functional genomics research. *Bioinformatics* **21**, 3674-3676 (2005).

4 Zdobnov, E. M. & Apweiler, R. InterProScan – an integration platform for the signature-recognition methods in InterPro. *Bioinformatics* **17**, 847-848 (2001).
